# Deep Temporal Organization of fMRI Phase Synchrony Modes Promotes Large-Scale Disconnection in Schizophrenia

**DOI:** 10.3389/fnins.2020.00214

**Published:** 2020-03-27

**Authors:** Tahereh S. Zarghami, Gholam-Ali Hossein-Zadeh, Fariba Bahrami

**Affiliations:** ^1^Bio-Electric Department, School of Electrical and Computer Engineering, College of Engineering, University of Tehran, Tehran, Iran; ^2^Human Motor Control and Computational Neuroscience Laboratory, School of Electrical and Computer Engineering, College of Engineering, University of Tehran, Tehran, Iran

**Keywords:** resting-state fMRI, instantaneous phase synchrony, functional networks, temporal hierarchy, metastate, trajectory, schizophrenia

## Abstract

Itinerant dynamics of the brain generates transient and recurrent spatiotemporal patterns in neuroimaging data. Characterizing metastable functional connectivity (FC) – particularly at rest and using functional magnetic resonance imaging (fMRI) – has shaped the field of dynamic functional connectivity (DFC). Mainstream DFC research relies on (sliding window) correlations to identify recurrent FC patterns. Recently, functional relevance of the *instantaneous phase synchrony* (IPS) of fMRI signals has been revealed using imaging studies and computational models. In the present paper, we identify the repertoire of whole-brain inter-network IPS states at rest. Moreover, we uncover a hierarchy in the temporal organization of IPS modes. We hypothesize that connectivity disorder in schizophrenia (SZ) is related to the (deep) temporal arrangement of large-scale IPS modes. Hence, we analyze resting-state fMRI data from 68 healthy controls (HC) and 51 SZ patients. Seven resting-state networks (and their sub-components) are identified using spatial independent component analysis. IPS is computed between subject-specific network time courses, using analytic signals. The resultant phase coupling patterns, across time and subjects, are clustered into eight IPS states. Statistical tests show that the relative expression and mean lifetime of certain IPS states have been altered in SZ. Namely, patients spend (45%) less time in a globally coherent state and a subcortical-centered state, and (40%) more time in states reflecting anticoupling within the cognitive control network, compared to the HC. Moreover, the transition profile (between states) reveals a deep temporal structure, shaping two metastates with distinct phase synchrony profiles. A metastate is a collection of states such that within-metastate transitions are more probable than across. Remarkably, metastate occupation balance is altered in SZ, in favor of the less synchronous metastate that promotes disconnection across networks. Furthermore, the trajectory of IPS patterns is less efficient, less smooth, and more restricted in SZ subjects, compared to the HC. Finally, a regression analysis confirms the diagnostic value of the defined IPS measures for SZ identification, highlighting the distinctive role of metastate proportion. Our results suggest that the proposed IPS features may be used for classification studies and for characterizing phase synchrony modes in other (clinical) populations.

## Introduction

Growing evidence suggests that large-scale functional connectivity (FC) is inherently *transient*. That is, functional networks reconfigure not only in response to task demands (Gonzalez-castillo and Bandettini, 2019) but also during resting state (Chang and Glover, 2010; Deco et al., 2013, 2017; Hutchison et al., 2013; Preti et al., 2017). Importantly, transient FC at rest has neuronal underpinnings, as evidenced by concurrent imaging and electrophysiological recordings (Tagliazucchi et al., 2012, 2015; Keilholz et al., 2013; Keilholz, 2014; Thompson et al., 2014) and emergent dynamics from computational models (Deco et al., 2011, 2017; Rabinovich and Varona, 2011; Cabral et al., 2014). Moreover, recent findings show that spontaneous FC patterns are quasi-periodically *recurrent* (Thompson et al., 2014), and their temporal organization is non-random (Deco et al., 2011; Vidaurre et al., 2017). Hence, dynamical organization of resting-state connectivity has been conceptualized as nonstop (and non-random) excursion through a bounded repertoire of metastable connectivity modes, called *states* (Ghosh et al., 2008; Deco et al., 2011; Cabral et al., 2017).

Neuroimaging furnishes non-invasive investigation of metastable brain phenomena, at multiple temporal and spatial scales. Specifically, functional magnetic resonance imaging (fMRI) is popular for its high spatial resolution and inherent circumvention of the inverse problem (i.e., source localization). As such, various techniques have been developed to capture regularities in the evolving spatiotemporal patterns of fMRI signals (Hutchison et al., 2013; Calhoun et al., 2014; Preti et al., 2017). Conventional dynamic FC (DFC) techniques track the statistical dependencies between neuroimaging series in successive epochs of data, which is known as the *sliding window* approach. Notably, window size has been a controversial parameter, posing a trade-off between the temporal resolution and statistical significance of the observed FC fluctuations (Hindriks et al., 2015; Leonardi and Ville, 2015; Zalesky and Breakspear, 2015). Meanwhile, a recent addition to the set of DFC measures is the instantaneous phase synchrony (IPS) of fMRI series, which eschews the window complications (Glerean et al., 2012) and has shown functional relevance in empirical and modeling studies.

Instantaneous phase synchrony is relatively new in fMRI (Laird et al., 2002; Deshmukh et al., 2004; Kitzbichler et al., 2009) despite being an established measure for analyzing electroencephalography (EEG), magnetoencephalography (MEG), and electrophysiological recordings (Tass et al., 1998; Lachaux et al., 1999, 2000). Once applied to fMRI signals, IPS reflects momentary phase alignment in the slow fluctuations of hemodynamic responses. Adopting IPS as a DFC measure can improve the temporal resolution of connectivity variations from the size of the window (typically 30–120 s) to the sampling time of the imaging modality (usually TR = 0.7–3 s).

Following evidence regarding the temporal variability of fMRI phase relationships within and between functional networks (Laird et al., 2002; Deshmukh et al., 2004; Kitzbichler et al., 2009; Chang and Glover, 2010; Glerean et al., 2012), verifying and characterizing the *spontaneous recurrence* of these patterns at rest and their functional relevance came into focus. The challenge is that, the instantaneity of IPS measures (between regions or networks) comes at the expense of noisy and irregular functional patterns, with spatiotemporal order that is not easy to capture. Early attempts relied on thresholding instantaneous phase couplings, converting them to binary patterns before further analysis (Kitzbichler et al., 2009; Ponce-Alvarez et al., 2015). In particular, Ponce-Alvarez et al. (2015) used non-negative matrix factorization (NNMF) to decompose a series of thresholded IPS matrices into the weighted sum of simpler (recurrent) components. This joint modeling and empirical study showed that phase interactions over a structurally plausible network give rise to recurrent synchronization patterns that resemble empirical arrangements – namely, the resting-state networks (Ponce-Alvarez et al., 2015). However, the threshold level (Δϕ = π /6) that defines phase synchrony had been chosen arbitrarily in this research. More recent studies (Cabral et al., 2017; Lord et al., 2019) avoid thresholding, but represent each bivariate IPS matrix by its first eigenvector, and apply (k-means) clustering on these summary vectors to identify recurring arrangements. This is also a practical approach that inevitably discards some of the phase coupling information, and still struggles with cluster validation (Lord et al., 2019). In short, finding natural order in IPS data has not been straightforward, despite certain simplifications.

Recently, conventional DFC^1^ studies have disclosed more sophisticated spatiotemporal order in resting-state data, which remains to be verified in the phase coupling realm. That is because, any connectomic order arising from correlation-based DFC analyses does not necessarily generalize to IPS dynamics, due to their inherently different mathematical properties and timescale, moderate association between their temporal variations and non-linear relationship on a topological level (in terms of time averages) (Pedersen et al., 2018). In the following, we review some prominent DFC findings, which have not been investigated using phase relationships and motivated the present analysis:

• Recent studies have revealed a *hierarchical organization* in the (non-random) temporal arrangement of functional states at rest (Vidaurre et al., 2017); i.e., temporal order beyond sample-to-sample transitions. Importantly, this slower temporal organization is heritable and related to personality traits (Vidaurre et al., 2017, 2019). Currently, we do not know whether the spontaneous recurrence of IPS patterns is similarly governed by a deep temporal arrangement.
• It was lately shown that the *trajectory* of connectivity evolution holds functionally relevant information, which becomes obscured in the usual cluster analyses (Miller et al., 2016). The IPS trajectory may also bear functionally relevant information, especially given its high (single-TR) temporal resolution.
• Functional integration is inherently multi-scale; that is, it coordinates neurons, micro-columns, and macroscopic regions (which form functional networks) and also organizes large-scale network interactions. Ongoing network interactions reflect higher-order functional organization in the brain, which is related to one’s character and cognitive performance (Vidaurre et al., 2019) and is modulated in various disorders (Du et al., 2018; Díez-cirarda et al., 2018; Gilbert et al., 2018; Rashid et al., 2018; de Lacy and Calhoun, 2019; Espinoza et al., 2019; Fu et al., 2019). So far, a good number of IPS studies have relied on (anatomical) atlases (Ponce-Alvarez et al., 2015; Cabral et al., 2017; Lord et al., 2019) or have investigated few functional networks (Chang and Glover, 2010); some have not been concerned with the recurrence of IPS modes (Pedersen et al., 2018; Zhang et al., 2019) or their recurrence characterizations have not been purely phase-based^2^ (Yaesoubi et al., 2015, 2017). Hence, current literature has not portrayed the repertoire of whole-brain inter-network IPS states based strictly on phase relationships – which could furnish new insights into the large-scale dynamic functional connectome. Notably, if large-scale phase couplings hold functional information – as speculated – they may also be modulated in neurological and psychiatric disorders.
• Schizophrenia^3^ (SZ) is a severe psychiatric disorder, which has affected some 23.6 million people worldwide by 2013, with a lifetime prevalence of about 1% (Murray et al., 2016). SZ is commonly known as a connectivity disorder^4^. In 1998, Friston (1998) proposed a *disconnection hypothesis* for SZ that stemmed from positron emission tomography (PET) findings. Ever since, fMRI has expedited the examination of disrupted functional organization of the brain in SZ (Liang et al., 2006; Fornito et al., 2012; Damaraju et al., 2014; Ma et al., 2014; Van Den Heuvel and Fornito, 2014). Dysfunctional connections within and across brain regions have been frequently reported in SZ studies^5^. Importantly, SZ disconnection affects between-network interactions as well, based on prior (correlation-based) DFC analyses (Damaraju et al., 2014; Rashid et al., 2014). It remains to be investigated whether instantaneous phase coupling of functional networks reflects disconnection in SZ, and whether whole-brain network-level IPS measures hold diagnostic value in this context.

In the present work, we try to address the above questions in an empirical study. Specifically, we analyze a publicly available SZ dataset while extending IPS characterization to higher functional and temporal levels. We hypothesize that disconnection in SZ relates to the spatial and (deep) temporal organization of network level IPS modes, and to the trajectory of large-scale phase coupling patterns, in this disorder. The results on SZ show the effectiveness of the proposed approach, which may be used in other applications as well.

## Materials and Methods

### Dataset and Pre-processing

We used the SZ dataset of the Center for Biomedical Research Excellence (COBRE) (Çetin et al., 2014). The dataset comprises of 72 SZ patients and 75 healthy subjects (18–65 years old). The patients had been diagnosed using the Structured Clinical Interview for DSM-IV Axis I Disorders (SCID-I) (First et al., 2002). Any subject with history of neurological disorder, mental retardation, severe head trauma, active substance abuse or dependence within the past year had been excluded from the study. Each participant was scanned at rest for 5 min on a 3-T Siemens Tim Trio scanner, and instructed to fixate on a central cross. A total of 150 (T2^∗^-weighted) functional volumes were acquired using a gradient-echo EPI sequence (TR = 2 s, TE = 29 ms, flip angle = 75°, 33 axial slices, ascending acquisition, matrix size = 64 × 64, voxel size = 3.75 × 3.75 × 4.55 mm, field of view = 240 mm). A high-resolution T1-weighted structural image was also collected for each subject.

Functional images were pre-processed in SPM12 software^6^ as follows: the first five volumes were discarded to allow for T1 equilibration; the remaining images were realigned (i.e., head motion corrected), slice-timing corrected, co-registered to the anatomical image of the subject, warped to the standard Montreal Neurological Institute (MNI) template (Collins et al., 1998), resampled to 3 *mm*^3^ isotropic voxels and smoothed with a Gaussian kernel (FWHM = 6 mm). Global signal was not removed (Murphy and Fox, 2017). Based on the motion realignment parameters, framewise displacement (FD) was computed as the total absolute head motion between consecutive time points, assuming 50-mm head radius for converting rotations to translations (Power et al., 2012). Nine subjects with maximum head translation exceeding 3 mm were removed; 12 patients with mean framewise displacement (MFD) above 0.7 mm were left out; and 5 normal females were excluded to match gender proportion. Hence, 119 subjects (68 healthy controls [HC]/51 SZ) were retained, whose demographics have been included in Table 1. Two-sample *t* tests on the maximum head translation, MFD, and age, plus chi-squared test on the gender proportion did not show significant difference between the two groups (uncorrected *p*-values = 0.78, 0.20, 0.82, and 0.16, respectively).

**TABLE 1:** Demographics and clinical information of the participants.

	**Number**	**Age**	**Female/male**
Healthy controls	68	35.4 ± 11.8	18/50
Schizophrenia patients	51	35.9 ± 13.4	8/43

### Spatially Constrained Spatial Independent Component Analysis

In order to achieve a refined functional parcellation of the brain, we used aggregate functional networks from Allen et al. (2014) as *spatial priors* to run (spatially) constrained spatial ICA (csICA) (Lin et al., 2010) on each subject. This approach has a number of advantages: (1) csICA respects spatial variability of the networks across subjects – unlike fixed network templates; (2) csICA optimizes independence of the ICs (i.e., the networks) at the subject level and simultaneously maintains their correspondence across the group; (3) csICA has superior performance^7^ compared to a number of other established (principal component/regression based) back-reconstruction techniques (Calhoun et al., 2001; Beckmann et al., 2009; Erhardt et al., 2011); (4) csICA allows for the propagation of aggregate networks to individuals who were not included in the original group analysis. This follows because the spatial priors of csICA need to be only “partially correct” in this method (Lin et al., 2010).

**FIGURE 1:**
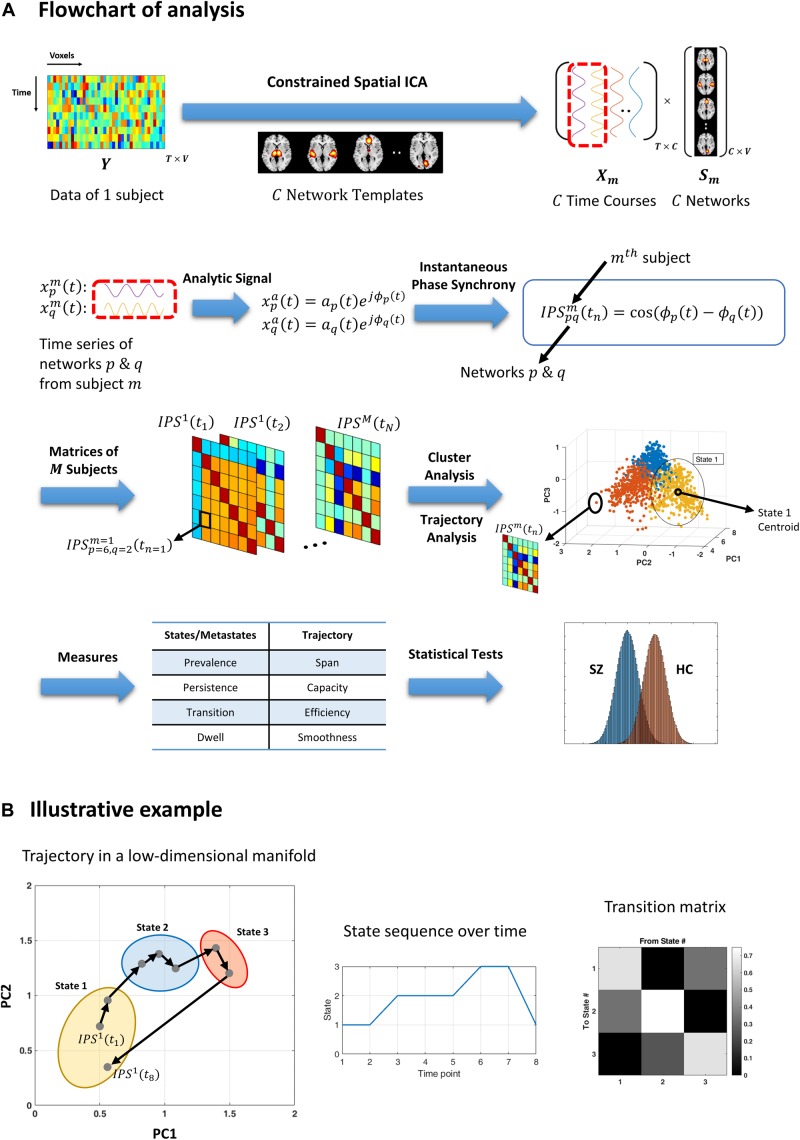
Flowchart of analysis and an illustrative example. **(A)** Resting-state fMRI data from each subject is decomposed using (spatially) constrained spatial independent component analysis (csICA) into C sub-networks (C = 50), based on the aggregate network templates from Allen et al. (2014). Time series corresponding to subject-specific networks are converted to complex analytic signals, from which instantaneous phases are extracted. Instantaneous phase synchrony (IPS) is computed as cosine of the difference between momentary phases, for each pair of networks and per subject. Time indexed IPS matrices of all subjects are clustered using spectral clustering. The three-dimensional plot (third row, right panel) shows an exemplar stack of IPS matrices projected on a (low-dimensional manifold, which is spanned by the first three principal components (PC). Cluster analysis assigns each data point (which is an IPS matrix) to one cluster centroid (i.e., a state). The color of each filled circle reflects the state label assigned to that IPS pattern, where only three states where assumed in this example. Trajectory analysis is conducted on the same IPS matrices (per subject), independently from cluster analysis. A number of measures are defined (in Table 2) to quantify the state and trajectory characteristics. These IPS measures are quantified and statistically compared between healthy controls (HC) and schizophrenia (SZ) patients. **(B)** An illustrative example highlighting the difference between cluster and trajectory analyses. Gray dots represent eight IPS matrices projected on their first two principal components. Arrows indicate temporal progression of the patterns, for a hypothetical session. The colorful ellipses correspond to three identified clusters. Hence, all the data points encompassed by one ellipse bear the same state label. The output of cluster analysis is a state sequence and a corresponding transition matrix (middle and right panels). Conversely, trajectory analysis focuses on sample-to-sample variations in the IPS patterns. For instance, the sum of the (L1) norms of all the black arrows constitutes the *trajectory length*. Also, the (L1) distance between the two farthest points is defined as the *span* of the trajectory. For the full list of IPS measures, please refer to Table 2.)

**FIGURE 2:**
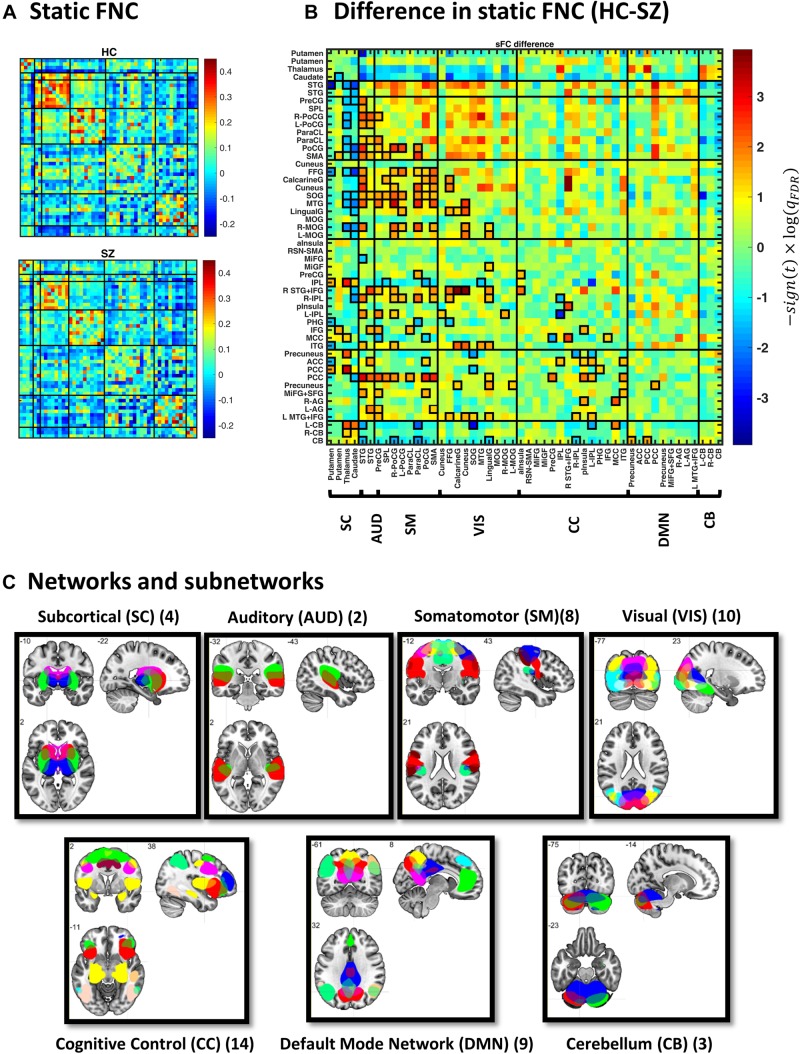
Static functional network connectivity (sFNC) analysis. **(A)** Average sFNC pattern for the healthy control (HC, upper panel) and schizophrenic (SZ, lower panel) group. Diagonal (unity) entries have been removed for better visualization. **(B)** Difference in sFNC (HC–SZ). The color bar denotes −10log(*q*_*FDR*_)×*sign*(*t*−*statistic*), following two-sample *t* test on Fisher *z*-transformed correlation values (*z* = atan*h*(*r*)). The 50 sub-networks and their organization into seven major networks have been reflected in the labels. Sub-network labels reflect the brain region with peak amplitude and refer to bilateral activations unless specified as left (L) or right (R). See Supplementary Figure S2 and Supplementary Table S1 in Allen et al. (2014) for the peak coordinates. **(C)** Spatial maps of sub-networks grouped into seven networks based on their anatomical and functional properties. Abbreviations: STG, superior temporal gyrus; PreCG, precentral gyrus; PoCG, postcentral gyrus; SMA, supplementary motor area; ParaCL, paracentral lobule; SPL, superior parietal lobule; MTG, middle temporal gyrus; FFG, fusiform gyrus; MOG, middle occipital gyrus; SOG, superior occipital gyrus; IPL, inferior parietal lobule; ITG, inferior temporal gyrus; MCC, middle cingulate cortex; pInsula, posterior insula; MiFG, middle frontal gyrus; IFG, inferior frontal gyrus; aInsula, anterior insula; PHG, parahippocampal gyrus; PCC, posterior cingulate cortex; AG, angular gyrus; ACC, anterior cingulate cortex; SFG, superior frontal gyrus; CB, cerebellum.

Hence, we adopted the 50 aggregate networks (estimated from a 100-component^8^ group spatial ICA analysis on 405 subjects in Allen et al. (2014), and implemented constrained spatial ICA (Lin et al., 2010) on the preprocessed data of every participant, using the Group ICA of fMRI Toolbox (GIFT^9^). Notably, the artifactual components (i.e., the physiological, head movement, and imaging artifact components) had already been identified and excluded in Allen et al. (2014), such that the remaining 50 functional parcels comprise sub-components of reproducible large-scale resting-state networks (Kiviniemi et al., 2009; Abou-Elseoud et al., 2010; Allen et al., 2011; Du et al., 2019). These networks include the subcortical (SC), auditory (AUD), somatomotor (SM), visual (VIS), cognitive control (CC), default mode network (DMN), and cerebellum (CB) (Figure 2C). For more details about the spatial maps of the networks and their peak coordinates, please see Supplementary Figure S2 and Supplementary Table S1 in Allen et al. (2014). For a schematic illustration of csICA please refer to the flowchart in Figure 1A.

**TABLE 2:** Measures of instantaneous phase synchrony (IPS).

**Measure**	**Description**
Prevalence	Probability of occurrence of each state
Persistence	Mean lifetime of each state (in seconds)
Transition Probability	Probability of switching from one state to another
Dwell	Probability of remaining in a given state

Trajectory length	Total L1 distance between successive IPS patterns
Span	Maximum L1 distance between IPS patterns
Capacity	Average L1 distance between IPS patterns
Efficiency	Ratio of Capacity to Trajectory Length
Smoothness	Average L1 similarity of successive IPS patterns

*The upper section contains measures based on cluster analysis. The lower section includes measures defined for the trajectory of IPS patterns. All indices are calculated per subject session.*

### Post-processing

The time courses associated with subject-specific ICs underwent additional post-processing to remove residual motion and artifactual sources of variation. That is, the time series were detrended to remove low-frequency scanner drift, orthogonalized with respect to the subject’s estimated motion parameters and their derivatives, and despiked to replace outlier points. Despiking was performed using AFNI’s 3dDespike algorithm which detects outlier time points (based on the median absolute deviation) and replaces them with interpolated values from a third-order spline fitted to the adjacent time points (Allen et al., 2014).

### Static Functional Network Connectivity

Average connectivity among the networks over the whole session was computed (for each subject) as the sample covariance matrix of the network time courses. Since resting-state FC is primarily shaped by low-frequency fluctuations of fMRI signals (Cordes et al., 2001), the network time series were bandpass filtered between (0.01 and 0.15) Hz (using a 5th-order Butterworth filter) before computing the static functional network connectivity (sFNC) matrices. Furthermore, sFNC was calculated for each group by averaging over their respective subjects. To highlight group differences, two-sample *t* tests were performed on each entry of the (Fisher *z*-transformed) sFNC matrices, and *p*-values were adjusted for multiple comparison using the false discovery rate (FDR) approach (Benjamini and Hochberg, 1995). Following convention, *q*_FDR_ < 0.05 was considered statistical significance.

### Instantaneous Phase Synchrony

The analytic representation of a real valued signal *x*(*t*) is a complex signal *x*_*a*_(*t*), with no negative frequency components. This complex signal can be constructed from the real signal using the Hilbert transform as follows (Boashash, 1992):

(1) $$x_{a}\,\left(t\right)=x\,\left(t\right)+j\,ℋ\,\left\{x\,\left(t\right)\right\}$$

where ℋ{.} denotes the Hilbert transform and *j* is the imaginary unit. The main property of analytic signal, x_a_(t), is that its Fourier transform is the same as that of the original real-valued signal, but only covers the positive frequencies. As a result, this complex (analytic) signal converts the original time series into two separate time series (which are the real and imaginary parts), from which useful aspects of the signal can be studied – such as instantaneous phase. In particular, for a *narrowband* signal expressed as *x*(*t*) = *a*(*t*)cos(ϕ(*t*)), the corresponding analytical representation is (Bedrosian, 1962):

(2) $$x_{a}\,\left(t\right)=a\,\left(t\right)\,\exp\left(j\,\phi \,\left(t\right)\right)$$

where *a*(*t*) and ϕ(*t*) are instantaneous envelope and instantaneous phase, respectively. Hence, for two real narrowband signals (*x*_*p*_(*t*) and *x*_*q*_(*t*)), the difference between their instantaneous phases reflects their phase synchrony. To get a normalized measure between −1 and +1, the cosine of the phase difference is considered (Glerean et al., 2012; Cabral et al., 2017):

(3) $${\mathrm{IPS}}_{\mathrm{pq}}\,\left(t\right)=\cos\,\left(\phi_{\text{p}}\,\left(t\right)-\phi_{\text{q}}\,\left(t\right)\right)$$

So, when the instantaneous phases of two signals fully align at time t, IPS(*t*) reaches its maximum value of +1; conversely, for 180° phase difference, this measure falls to −1, reminiscent of the familiar correlation and anti-correlation notions (herein called coupling and anticoupling).

The post-processed network time courses in our study were bandpass filtered in the 0.01–0.08 Hz range (Cabral et al., 2017) using Parks-McClellan linear-phase finite impulse response filter (Mcclellan et al., 1973; Glerean et al., 2012). The corresponding analytic signals were used to compute instantaneous phase series (Eqs. 1 and 2). Subsequently, IPS was calculated for each pair of networks (*p* and *q*) at each time point (using Eq. 3) to construct time-dependent IPS matrices (Figure 1A). These phase coupling matrices reflect the momentary phase similarities of *C=50* resting-state (sub)networks, for each subject. The outstanding task was to characterize the recurrence of these IPS patterns.

### Identifying Recurrent IPS Patterns

In order to identify the main modes of phase coupling, across time and subjects, we used cluster analysis. Clustering relies on some measure of (dis)similarity between the samples, which are symmetric IPS matrices in this context. A common measure of similarity is *cosine similarity*:

(4) $$\mathrm{cosine}\,\mathrm{similarity}\,\left(\vec{{\mathrm{IPS}}^{m}}\,\left(t_{i}\right),\vec{{\mathrm{IPS}}^{n}}\,\left(t_{j}\right)\right)=\frac{\vec{{\mathrm{IPS}}^{m}}\,\left(t_{i}\right)\cdot \vec{{\mathrm{IPS}}^{n}}\,\left(t_{j}\right)}{||\vec{{\mathrm{IPS}}^{m}}\,\left(t_{i}\right)||\,||\vec{{\mathrm{IPS}}^{n}}\,\left(t_{j}\right)||}$$

where $\vec{I\,P\,S^{m}}\,\left(t_{i}\right)$ is a vector holding the unique (upper or lower triangular) entries in the symmetric IPS matrix of the *m*^*th*^ subject, at time *t*_*i*_; the dot in the numerator stands for dot product and ||⋅|| is the magnitude (i.e., Euclidean norm). We work with a related measure called *angular similarity*, defined as follows:

(5) angular⁢similarity=1-angular⁢distance

(6) $$\mathrm{angular}\,\mathrm{distance}=\frac{\cos^{-1}\left\{\mathrm{cosine}\,\mathrm{similarity}\right\}}{\pi }$$

Angular similarity varies between 0 and 1, and this positivity is useful for constructing an unsigned graph^10^ (to perform spectral clustering, in what follows). Moreover, angular distance satisfies the triangle inequality (unlike cosine distance) and qualifies as a proper distance metric. Note that we have not performed data reduction on the phase coupling matrices prior to (angular) similarity computation, in order to retain maximal information about the phase synchrony patterns.

**FIGURE 3:**
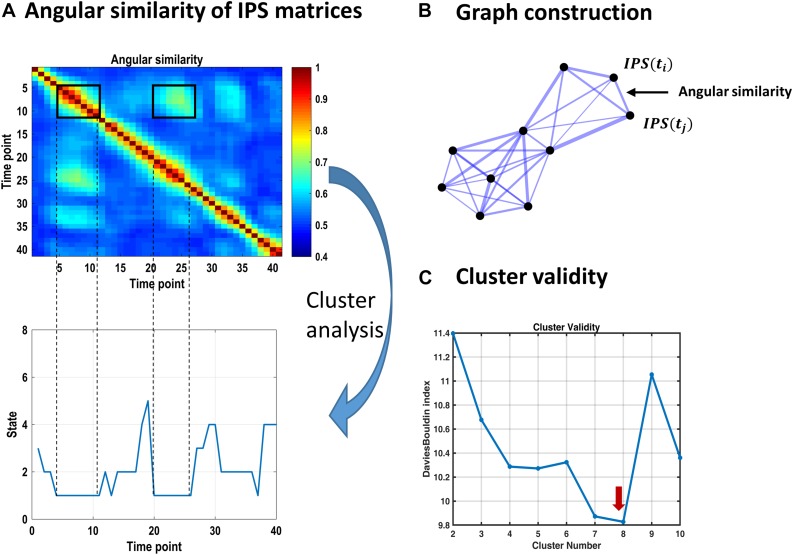
Identifying recurrent IPS patterns through cluster analysis. **(A)** Top plot shows a representative portion of the (symmetric) angular similarity matrix for 40 consecutive IPS patterns. That is, each entry denotes the angular similarity between two time-indexed IPS matrices (Eq. 5). Recurrent transient patterns are automatically placed in the same cluster, following spectral clustering. For instance, IPS patterns realized between time points 5 and 11 (enclosed by a black square on the diagonal) are highly similar to each other and to the patterns emerging over time samples 20–26. Hence, IPS matrices from both periods identify with the same cluster number (i.e., state 1), as illustrated in the lower panel. **(B)** Schematic of an (undirected weighted) graph associated with an angular similarity matrix. Each node corresponds to a time-indexed IPS matrix, and edge thickness reflects angular similarity. Using spectral clustering, this graph can be optimally divided into well-connected groups of nodes that are plausibly separated from each other, comprising the clusters of interest. **(C)** Cluster validity is established using the Davies–Bouldin index (which is the ratio of within-cluster scatter to between-cluster distance) quantified for a range of 2–10 clusters. The lowest value of this index reflects the most plausible clustering solution, which is eight for our data. The associated cluster centroids are illustrated in Figure 4.

As such, for *M* subject sessions, each comprising *N* time samples, an *MN*×*MN* (angular) similarity matrix was computed. A representative portion of this matrix has been illustrated in Figure 3A. From this similarity matrix, a (weighted undirected unsigned) graph was constructed, which has been schematically depicted in Figure 3B. Graph representation furnishes the use of concepts and tools from graph theory. In particular, *spectral graph theory* is the mathematical field of studying graph properties from the eigenvectors and eigenvalues of their associated adjacency and Laplacian^11^ matrices. For instance, optimally partitioning a graph into a number of sub-graphs is an NP-hard problem for which *spectral clustering* has offered computationally tractable (approximate) solutions (Shi and Malik, 2000; Luxburg, 2007).

**FIGURE 4:**
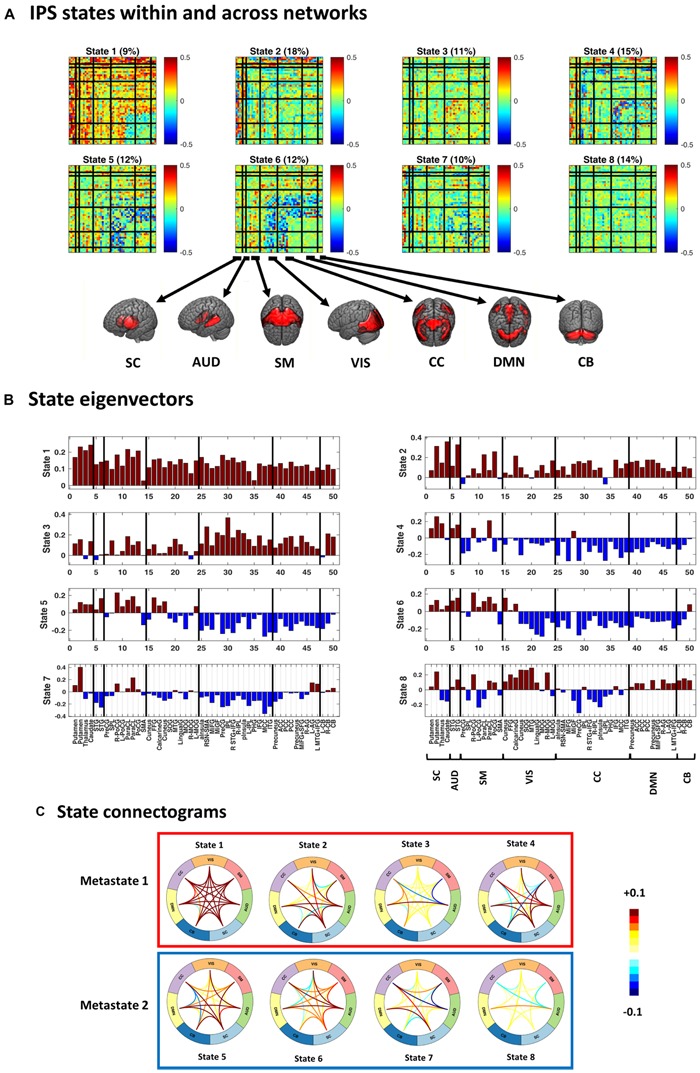
Instantaneous phase synchrony (IPS) states, in three representations. **(A)** Eight cluster centroids, depicted in matrix format. Each centroid stands for an IPS state. The relative expression of each state (across time and subjects) is printed above it. Surface colors reflect IPS values (Eq. 3). Matrix representation offers a detailed account of the phase coupling structure of each state, within and across seven functional networks. **(B)** The first eigenvector of each state is presented as a bar plot. Positive and negative entries constitute two detached communities. **(C)** State connectograms are concise representations of average inter-network phase couplings, where the average is taken over sub-components of each network. For instance, the central role of the subcortical (SC) network in state 2 is readily recognized by noting the warm colored arcs extending from SC to other networks. SC, subcortical; AUD, auditory; SM, somatomotor; VIS, visual; CC, cognitive control; DMN, default mode network; CB, cerebellum. For the sub-network abbreviations, please refer to the caption of Figure 2.

We used the spectral clustering algorithm of Ng et al. (2002). In this method, a low-dimensional representation of the pairwise similarity matrix is constructed from the *k* smallest eigenvectors^12^ of the normalized graph Laplacian matrix. These vectors constitute the columns of a new matrix, whose (normalized) rows serve as the feature vectors for a clustering algorithm. We used Gaussian mixture model (GMM) at this stage. To establish the optimal (*k*) number of clusters, the same procedure was repeated for *k* = 2–10 components and Davies–Bouldin index^13^ (Davies and Bouldin, 1979) was used to score cluster validity (Figure 3C). The optimal number of components (8 in this case) was selected and the resultant cluster centroids comprised the dominant *states* of inter-network phase coupling, across time and subjects (Figure 4A). Thereafter, each time-indexed IPS matrix was assigned to the most plausible centroid (i.e., state) based on its posterior probability of assignment (which is called hard assignment in the clustering literature).

.

### Characterization of IPS Transient Behavior

Following hard/crisp clustering, every IPS matrix (at each time point) bears one state label ∈ {1:8}. The resultant sequence of states per subject (see Figure 3A, lower panel) was used to characterize the transient expression of phase synchrony modes over time. Specifically, we call the fraction of IPS patterns assigned to each state the *prevalence* of that state. Other properties of interest are the probability of *transition* between states, the probability of remaining in a particular state (called *dwell*), and the average period over which a state is held continuously (called *persistence*). These measures are summarized in Table 2. They were computed per subject and compared between (HC and SZ) groups using permutation-based two-sample *t* tests, followed by FDR correction for multiple comparisons.

We also investigated the relationship between the transition probability and the similarity of states. It has been suggested that transition probability between states is related to their connectomic similarity; i.e., the brain undergoes smooth connectivity changes over time (Vidaurre et al., 2017). Specifically, a significant positive correlation between the connectomic similarity of (correlation-based) functional states and the transition probability between them has been reported (Vidaurre et al., 2017). We intended to test the same hypothesis about phase synchrony modes and their transition profile. We tested, additionally, for any correlation between transition probabilities and state prevalence (and persistence) similarities. The correlation values are reported alongside 95% confidence intervals (CIs), which were derived from 1000 bootstrap resamplings of the subjects.

### Deep Temporal Organization of Phase Synchrony Modes

Recent DFC research has revealed a hierarchy in the temporal organization of connectivity states at rest (Vidaurre et al., 2017). In other words, the repertoire of connectivity modes comprises of groups of states (called *metastates*) such that within-metastate transitions are more probable than across. So, once the brain enters a metastate, it is more likely to circulate within that community of states for a while, before switching into another metastate. This speaks to a separation of temporal scales, i.e., a temporal hierarchy. That is to say, the dynamics of the metastates and states unfold on slower and faster timescales, respectively. So far, this deep temporal organization has been endorsed for correlation-based connectivity modes.

Similarly, to examine whether the temporal arrangement of phase synchrony states conforms to a hierarchy, we looked for communities within the transition matrix. By regarding the transition probabilities as adjacency values, the transition matrix can be treated as an adjacency matrix, with an associated graph. Due to the small number of states (i.e., 8), we looked into the coarsest division of the transition matrix (into two partitions). In spectral graph theory, bi-partitioning can be achieved using the Fiedler vector (which is the second smallest eigenvector) of the graph Laplacian (Fiedler, 1973), using the sign of the corresponding vector entries. In other words, positive and negative entries of the Fiedler vector determine how the graph nodes (here states) can be placed into two maximally disjoint partitions. This is an approximate solution to the minimum-cut partitioning problem on graphs (Riolo and Newman, 2014). The result was validated using hierarchical clustering. The presence of distinct well-connected communities in the transition matrix would demonstrate temporal order beyond sample to sample switching. If plausible metastates exist, the remaining task would be investigating their phase synchrony profiles and temporal characteristics – plus potential alterations in SZ.

### Trajectory of Phase Coupling Evolution

Cluster analysis is a useful approach for capturing the overall composition and transience of phase couplings patterns. With the usual crisp assignment, all the IPS matrices that belong to a particular cluster collapse onto one dimension (which is the state label), disregarding individual differences and proximity to the center or boundary of the cluster. Previous DFC work (Miller et al., 2016) has shown that this crude dimensionality reduction overlooks information in the *trajectory* of connectivity and could over/underestimate connectomic variations.

To circumvent this issue, Miller et al. (2016) have reparameterized time-dependent connectivity patterns by discretizing their probabilistic assignments to a few prototypical states (derived from data decomposition). In other words, they have projected time-dependent connectivity patterns on (five) representative connectivity modes and discretized the resultant coefficients; thus, summarizing each connectivity pattern with a five-element integer vector. This method reveals valuable trajectory information and is more indicative of time to time connectivity differences than hard (crisp) state assignment. However, this approach still depends on data decomposition, followed by projection and discretization, and the FC patterns are derived from signal correlations, over successive windows. Instead, we intended to follow the trajectory of IPS patterns, in a high-dimensional space and at every time point, without resorting to decomposition and discretization. Hence, we defined a number of intuitive measures to characterize the evolution of phase couplings. In the following, we describe these measures.

Due to the high-dimensional nature of the IPS matrices (with 50×49/2 = 1225 unique features), L1 norm^14^ is a more effective dissimilarity measure than L2 (Euclidean) distance (Aggarwal et al., 2001). So, we computed *trajectory length* as the sum^15^ of L1 distances between successive IPS matrices, per subject session; hence, trajectory length = $\sum_{i=1}^{N-1}L_{i\,\left(i+1\right)}^{m}$. Plus, the maximum L1 distance (over all IPS patterns in a session) was taken as the *span* of the trajectory: span = $\max_{i,j}L_{i\,j}^{m}$; *i*,*j* ∈ {1:*N*}. Although informative, this maximum value does not reflect dispersion of the realized IPS patterns over the hyperspace (see schematic trajectory in Figure 1B). Hence, by averaging over all pairwise L1 distances (per subject) we computed the *capacity* of the trajectory; i.e., capacity = $<L_{i\,j}^{m}>$. Furthermore, the trajectory *efficiency* was defined as the ratio of capacity to trajectory length, for each subject. In this sense, an efficient trajectory traverses a large portion of the state space (i.e., realizes diverse patterns) with relatively small steps; conversely, a very inefficient trajectory is a long one (with large misplanned steps) that eventually remains confined in a small hyperspace.

Another useful feature is the *smoothness* of the trajectory. Smoothness was defined as the average of L1 similarities between consecutive IPS patterns (per subject), where L1 similarity stands for the reciprocal of L1 distance; hence, smoothness = $\frac{1}{N-1}\,\sum_{i=1}^{N-1}{\left(L_{i\,\left(i+1\right)}^{m}\right)}^{-1}$. Using these measures, we looked for potential modulations of the phase coupling evolution in the patient group. For a summary of the measures defined in this section please refer to the lower section of Table 2.

### Phase Synchrony Features as SZ Predictors

We used the phase synchrony measures, introduced in the previous sections, to predict the SZ label using a linear regression model. Age, gender, and MFD were considered as potential confounds. The model was set up as follows:

$$y_{\mathrm{diagnosis}}=\beta_{0}+\beta_{\mathrm{measure}}\,X_{\mathrm{measure}}+\beta_{\mathrm{age}}\,X_{\mathrm{age}}+\beta_{\mathrm{gender}}\,X_{\mathrm{gender}}$$ (7)

where *y*_*diagnosis*_ is a binary vector with SZ diagnosis coded as 1 and HC as 0. The linear relationship between each IPS measure (*X*_*measure*_) and the diagnosis label (*y*_*diagnosis*_) was inspected separately. Moreover, an *F* test was also conducted on each regression model to assess the significance of the linear relationship. *p*-values were FDR corrected and significance corresponds to *q* < 0.05. Hence, for each measure, we report the adjusted *R*^2^, β_*measure*_, and *q*_FDR_ values.

### Validation Analyses

#### Subject-Specificity of Networks

To investigate individual differences in the estimated ICs and their correspondence to the aggregate networks, we followed the validation procedure in Lin et al. (2010). Namely, subject components were variance normalized and a voxel-wise one-sample *t* test was performed on each of the 50 components, across subjects. Each *t*-map was subsequently thresholded at an FDR corrected *q* < 0.01. The normalized spatial correlation of subject-specific (FDR-corrected) IC maps with the corresponding IC templates was computed. Average correlation for each subject, over all ICs, was in the (0.6–0.74) range. These values reflect high correspondence of individual networks to the templates [when compared with the (0.43–0.63) range in Lin et al. (2010)], as well as considerable amount of inter-subject variability. Notably, (normalized) spatial correlation to the template was always above 0.45 for any subject-specific IC, averaged around 0.69, and reached as high as 0.88. These results have been demonstrated in Supplementary Figure S1.

#### Genuineness of IPS Modes

To verify that the identified IPS patterns reflect genuine phase arrangements, the same cluster analysis was repeated on surrogate data. Relevant literature recommends phase shuffling (Theiler et al., 1992; Schreiber and Schmitz, 2000; Sun et al., 2012) and random circular shifting (Politis and Romano, 1992; Glerean et al., 2012) for generating surrogates. The former shuffles the phase spectra of the original time series while keeping the amplitude spectra unchanged. The latter applies a random circular shift to each of the original series, to disrupt the existing synchrony between them, while temporal dependencies within each series are preserved. We used both approaches and generated two sets of surrogates. If the clusters in the original dataset reflect meaningful and recurrent phase synchrony modes, they should be distinct from patterns identified from surrogate data.

#### Motion Effect

To inspect whether head motion is related to the emergence of any state, we investigated the correlations between state occurrences and concomitant FDs. Moreover, we checked for systematic differences between FDs associated with different states. That is, we computed average FD for each state, per subject: ${\bar{F\,D}}^{s}\,\left(i\right)$, where **s** is the state label and *i* is the subject index. Then, we conducted a one-way repeated-measures ANOVA to test for difference in the means of these state-dependent average-FDs.

#### Drowsiness Effect

A prior DFC study (Allen et al., 2014) has reported one FC state associated with drowsiness or light sleep. This speculation was based on the increasing occurrence rate of that state over time. To inspect this possibility, we fitted a line to the occurrence rate (in five-sample windows) of each state over time, for each subject. The slopes of the fitted lines were examined for potential non-zero trend using one-sample *t* test. If a state is associated with drowsiness, its occurrence rate is likely to show a positive trend over the length of the scan.

#### Smoothing Kernel Effect

To inspect sensitivity of the findings to the width of the smoothing kernel (in pre-processing), we repeated our analysis for data smoothed using a Gaussian kernel with FWHM = 9 mm. Recent research suggests that kernels spanning 2–3 voxels are optimal for fMRI preprocessing prior to ICA analysis at the subject level (Chen and Calhoun, 2018). From this validation analysis, we found that our key results (i.e., the nature of the states and metastates and their temporal profiles, as well as the IPS trajectory features and their alterations in SZ) are robustly replicated even with this wider kernel. The results have been provided as Supplementary Material.

## Results

### sFNC Indicates Disconnection in SZ

Figure 2A illustrates group-specific sFNC patterns, obtained for each subject and then averaged over each group. The average patterns reveal well-known modular organization within sensory systems and default mode components, as well as anticorrelation between them (Fox et al., 2005; Chang and Glover, 2010; Shirer et al., 2012). Group difference in sFNC (HC–SZ) is demonstrated in Figure 2B as −10log(*q*_*FDR*_)×*sign*(*t*−*statistic*). Significant differences have been marked in bold squares in the lower triangular part (corresponding to *q*_FDR_ < 0.05). From 1225 unique sFNC entries, 18% are significantly different between the two groups (13% have higher mean value in the HC group and 5% are higher in SZ). Specifically, stronger correlation among sensory areas of normal subjects as well as pronounced subcortical-sensory anticorrelation (compared to SZ patients) is in accordance with previous findings (Damaraju et al., 2014). Moreover, a two-sample *t* test on the ensemble of the matrix entries showed that sFNC is globally stronger in normal subjects (*p* < 1e−4).

### Recurrent IPS States Comprise Distinct and Diverse Patterns

The representative portion of the angular similarity matrix in Figure 3A indicates that phase coupling evolution is gradual (hence the heavy diagonal). Moreover, similar patterns emerge over non-adjacent epochs, creating the bright off-diagonal patches. The goal of cluster analysis on IPS patterns was to quantify this recurrence. Using spectral clustering, eight distinct IPS arrangements were identified, which recur over time and across subjects. By associating each IPS matrix with one state label, a sequence of states was formed (see Figure 3A, lower panel). The overall prevalence of each state has been imprinted on top of the corresponding matrix in Figure 4A.

Figure 4 shows the cluster centroids (i.e., the IPS states) in three formats: matrices, eigenvectors, and connectograms. Clearly, the states differ in their functional architectures. State 1 denotes high level of phase synchrony within and across most networks, while parts of DMN, CB, and CC are decoupled from each other. In state 2, SC and AUD show strong (anti)coupling with respect to the rest of the networks. State 3 reflects notable phase coupling within and across CC, DMN, and CB. In state 4, SC and AUD have strong synchrony with other components while SM, VIS, CC, DMN, and CB are mostly anticoupled or decoupled. State 5 shows an internally incoherent CC, which is also anticoupled to DMN and CB, while DMN is internally integrated. In state 6, (parts of) VIS and CC are anticoupled within and across each other and have mixed (coupling and anticoupling) relationship to DMN and CB. State 7 is noted for strong SC connections, disintegrated CC, and anticoupling of AUD to SM and CC. State 8 is a decoupled state on average, with faintly more synchronous VIS and slightly anticoupled CC. Note that the (unity) diagonal values have been removed from all matrices to improve image contrast.

Figures 4B,C provide alternative useful representations of the states. Bar plots in Figure 4B illustrate the first eigenvectors of the states. Hence, the complex coupling and anticoupling relations in each state make up the detached modes in the leading eigenvectors (Bonacich and Lloyd, 2004; Bonacich, 2007; Cabral et al., 2017). The connectograms in panel C show average across-network phase couplings, which serve as concise representations of large-scale IPS modes. Of note is the variety of the patterns identified from empirical data. We will see (in the Results section) that these functional architectures are distinct from accidental phase synchrony arrangements obtained from surrogate data.

Figure 4 also shows that, averaged over all subjects, empirical state 1 is the least common (with 11% prevalence) while state 2 is visited most often (18% prevalence). In the next section, we will inspect group-specific results and show that SZ state proportion is profoundly different from that of the HC.

### IPS State Proportion Has Been Altered in SZ

**FIGURE 5:**
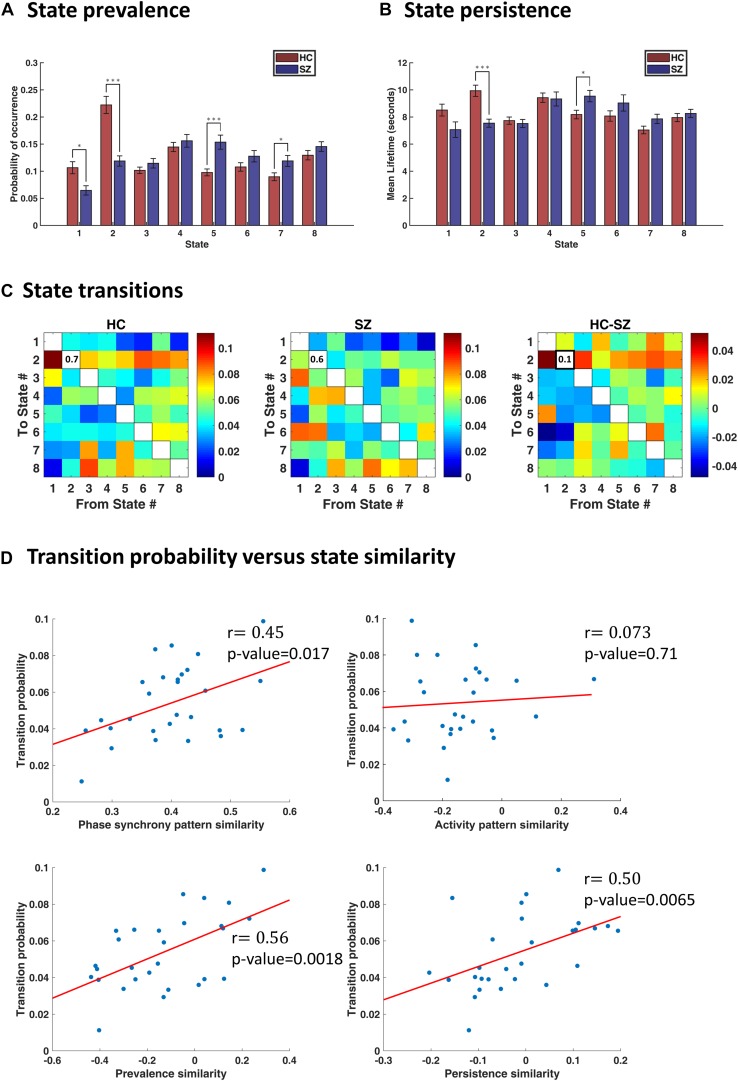
Characterization of transience in IPS states. **(A)** State prevalence (i.e., probability of occurrence) for each state, in the healthy control (HC) and schizophrenic (SZ) group. Error bars denote standard errors of mean (SEM). Group differences were assessed using permutation-based *t* tests. States 1 and 2 are more frequently visited by the HC, whereas states 5 and 7 are more prevalent in the patient group. **(B)** Persistence (i.e., mean lifetime) of each state, per group. The HC remain longer (continuously) in state 2, whereas SZ subjects prefer to linger in state 5. **(C)** Group-average transition patterns and their difference (HC–SZ). Diagonal values have been removed to improve image contrast. Only one entry (i.e., the probability of dwell in state 2) is significantly different after FDR correction. **(D)** Relationship between transition probability and state similarity. State similarity is assessed from four aspects: IPS pattern similarity, activity map similarity, prevalence similarity, and persistence similarity. Activity map resemblance is uncorrelated with transition probability between states; however, similarity in the other three functional and temporal aspects of the states significantly correlates with the probability of switching between them. The correlation coefficient (*r*) is imprinted beside each plot, and red lines denote best fitted lines. In panels **(A,B)**, asterisks show statistically significant differences, after FDR correction (^∗^ indicates *p* < 0.05; ^∗∗∗^ indicates *p* < 0.001).

By inspecting state prevalence values in both groups (Figure 5A), we note that patients express the first two states almost half as much as normal subjects (6% vs. 11% for state 1, and 12% vs. 22% for state 2). Instead, patients would rather spend more time in state 5 or 7 (15% vs. 10% for state 5, and 12% vs. 9% for state 7). Importantly, the first two states are more globally connected than others (see Figure 4B, the leading eigenvectors), while state 5 is most notable for anticoupling within CC and negative coupling of CC to DMN and CB. State 7 also reflects de/anti-coupling among CC components. Corroborating these results, the persistence plot (in Figure 5B) shows that patients tend to remain in state 5 about 1.2 s longer than healthy subjects, on average; conversely, SZ patients cannot hold state 2 as long as the HC (persistence of state 2 = 7.5 s and 10 s, for SZ and HC, respectively). These differences were significant after FDR correction and show that the proportion and average duration of IPS states have been altered in SZ. Notably, this alteration favors less synchronous states that lack a cohesive cognitive control network.

In addition to these state occupation features, the switching pattern between states is another informative aspect of IPS transience, which we will examine in the next section.

### IPS State Transitions Show Minimal Change in SZ

In Figure 5C, we have shown group-specific transition matrices and their difference (HC–SZ). Although permutation tests on the transition probabilities returned a few significant uncorrected *p*-values (for transitions from/into states 2 and 5), none of them survived FDR correction, except the probability of dwell in state 2 (*q*_FDR_ < 0.05. This probability is on average 0.7 for the HC group and 0.6 for SZ patients. This result is in line with our previous finding that state 2 is almost twice as prevalent in healthy subjects (compared to the patients) and persists longer (Figures 5A,B).

Although the transition probabilities are not very informative for distinguishing patients from controls (in the present study), they still reveal important information about the spontaneous spatiotemporal reorganization of the brain, in general. We will demonstrate this in the following.

### State Transition Probability Correlates With State Similarity

Previous research has suggested that the transition probability between connectivity modes correlates with the similarity of the corresponding connectivity patterns; that is, connectivity changes occur rather gradually over time (Vidaurre et al., 2017). However, the recurrent functional states in Vidaurre et al. (2017) reflect correlations. We tested the same hypothesis about fMRI instantaneous phase coupling variations. That is, we correlated the transition probabilities with the IPS state similarities. Notably, we assessed similarity of the states from both functional and temporal aspects.

To quantify functional similarity of the states, pairwise correlations were computed between the eight IPS states, over all subjects, resulting in an 8×8 symmetric state similarity matrix. Correspondence of this similarity matrix to the average transition matrix was quantified using correlation analysis. The same procedure was repeated using prevalence similarity, persistence similarity, and activity similarity of the states.

The results (in Figure 5D) show that, indeed, similar states are more likely to follow each other in time. Phase synchrony resemblance of the states significantly correlates with the transition probabilities [*r* = 0.45, *p*-value = 0.017, 95% CI = [0.31, 0.57]). The temporal similarity of the states, namely, their prevalence and persistence associations, are also linearly related to their transitional behavior. Specifically, for prevalence similarity vs. transition probability: *r* = 0.56, *p* = 0.0018, 95% CI = [0.47, 0.71]; and for persistence similarity against transition probability: *r* = 0.50, *p* = 0.0065, 95% CI = [0.45, 0.72]. However, activity pattern resemblance is not correlated with transition probability (*r* = 0.073, *p* = 0.71, 95% CI = [−0.25, 0.46]), corroborating the results in Vidaurre et al. (2017). In other words, it is the connectivity (be it amplitude dependencies or phase coupling) structure that evolves with more self-consistence over time – rather than the activity profile.

As noted, the transition matrix holds abundant information about the spatiotemporal organization of the brain at rest. In the next section, we will go through the results of our metastate analysis – again based on the transition pattern – which speaks to higher-order temporal structure in the phase coupling variations.

### Metastates Induce Two Distinct Phase Synchrony Modes

**FIGURE 6:**
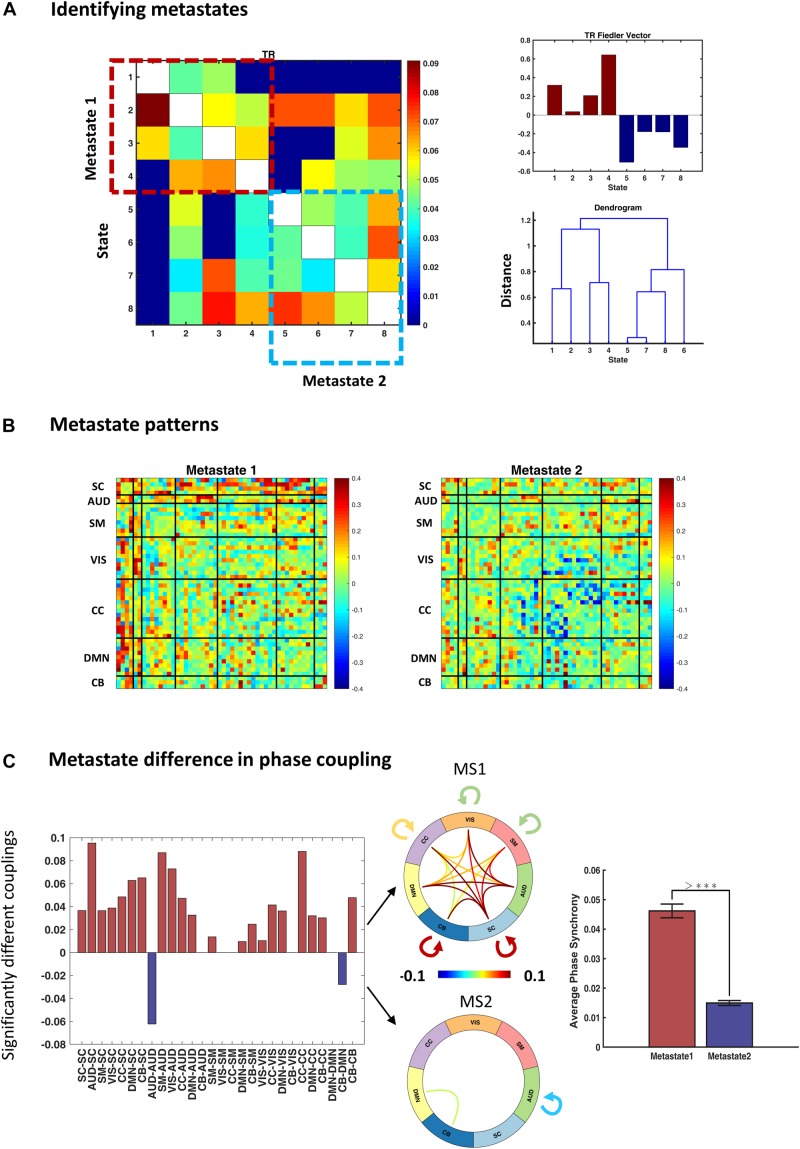
Identifying and characterizing metastates (MS). **(A)** Metastates are groups of states with higher probability of within-group transitions, than across. States 1–4 and 5–8 show this property, as evidenced by diagonal blocks on the group transition matrix. Entries of the transition matrix (left panel) denote 0.6th quantiles, across subjects. The top right panel shows the Fiedler vector (i.e., the second smallest eigenvector of the graph Laplacian associated with the transition matrix) and the lower panel contains the dendrogram of hierarchical clustering (cophenetic correlation coefficient = 0.22). Both methods acknowledge the same metastates. **(B)** Average metastate patterns, across subjects. MS1 is clearly more coherent (than MS2) and MS2 is notable for anticoupling of the cognitive control network components. **(C)** Difference in the phase coupling pattern of the metastates. Left panel: connections that are on average stronger in MS1 (or MS2) were identified using permutation-based paired *t* tests. Positive (pink) bars show (average) phase couplings that are significantly higher in MS1 than MS2; negative (blue) bars denote the inverse. The two connectograms (middle panel) convey the same information graphically. The bar plot (on the right) shows that average phase coupling is significantly higher in MS1, than MS2 (permutation-based paired *t* test: *p* < 1e–4). Error bars stand for standard errors of mean, and >*⁣** indicates *p* < 1e–4.

To identify metastates (MS), we treated the average transition pattern as an adjacency matrix and bi-partitioned the associated graph using the Fiedler vector (as elaborated in the “Methods” section). Figure 6A (right panel) shows the Fiedler vector alongside the hierarchical clustering dendrogram. Both approaches show that states 1–4 and 5–8 constitute two plausible partitions, i.e., metastates. The blocks on the main diagonal of the transition matrix (Figure 6A, left panel) show that within-metastate transitions are more probable, than across. For the sake of presentation, each entry of this matrix denotes 0.6th quantile of the corresponding transition probabilities, across subjects.

The average MS profiles are illustrated in Figure 6B. MS1 is clearly more coherent, and both SC and AUD networks have central roles in its functional architecture. Conversely, MS2 has lower overall phase synchrony level (Figure 6C, bar plot) and shows a notable breakup and anticoupling among CC components. We tested for connectomic differences between these MSs using permutation-based paired *t* tests. The results in Figure 6C show that out of ($\frac{7\times 6}{2}+7=$) 28 unique connections within and across the main (seven) networks, 21 couplings are on average stronger in MS1, only 2 connection means are higher in MS2, while 5 connections are not significantly different (after FDR correction). The connectograms in Figure 6C show the mean values for the statistically different connections in the two MSs. Moreover, the bar plot (right panel) demonstrates that global phase coupling of MS1 is significantly higher than MS2 (*p*-value < 1e−4). Having characterized the metastates, the outstanding task is to verify whether the temporal organization of these MSs has been altered in SZ.

### The Less-Synchronous Metastate Dominates in SZ

**FIGURE 7:**
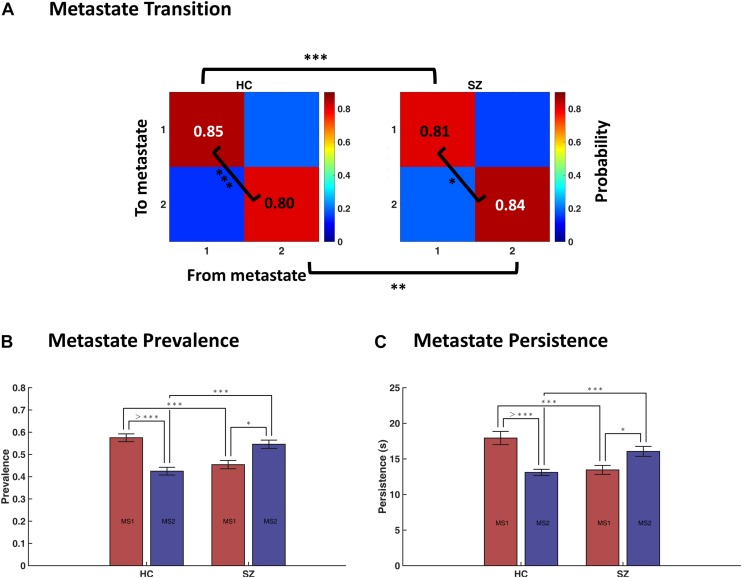
Temporal characteristics of metastates (MS). **(A)** Average transition probability matrices of metastates, for the healthy control (HC, left panel) and schizophrenic (SZ, right panel) subjects. Dwell probabilities (i.e., the diagonal entries) were compared within and between groups. **(B)** Difference in the prevalence (i.e., probability of occurrence) of MS1 and MS2, within and across groups. **(C)** Difference in the persistence (i.e., uninterrupted occupancy) of metastates, within and across groups. The key finding here is that MS1 is dominant in the HC, whereas MS2 prevails in SZ. Statistical tests were permutation-based *t* tests (paired, when appropriate). Error bars denote standard errors of mean (SEM). Asterisks denote significant difference in mean, after FDR correction (^∗^ indicates *p* < 0.05; ^∗∗^ indicates *p* < 0.01; ^∗∗∗^ indicates *p* < 0.001; > *⁣** indicates *p* < 0.0001).

We quantified the temporal profile of the metastates by computing their transition probabilities, as well as their prevalence and persistence values, in each group. Figure 7A shows that the probability of dwell in MS1 is higher in HC compared to SZ (0.85 versus 0.81, *p*-value < 1e−3). Conversely, the probability of dwell in MS2 is higher in SZ than HC (0.84 versus 0.80). Transition probabilities (from MS1 to MS2 and vice versa) are the complements of dwell values, so they are also significantly different between the two groups. Moreover, the dwell probabilities are significantly different *within* each group; that is, MS1 dwell is higher than MS2 dwell in HC subjects (*p* < 1e−3), and the opposite is true for SZ patients (*p* = 0.0162). All *p*-values were FDR corrected. These results become clearer as we inspect other temporal characteristics of the metastates.

Figures 7B,C show that MS1 is more prevalent and persists longer in the HC, compared to the SZ group (compare the pink bars); the inverse is true for MS2 (inspect the blue bars). All FDR-corrected *p*-values remained below 0.001 for these tests. But, more importantly, this plot is saying that the balance of MS expression for a typical SZ patient is the inverse of what is expected in a normal subject. That is, while MS1 is dominant in the HC (both in terms of prevalence and persistence), MS2 is the leading phase coupling mode in SZ. These within-group effects were investigated using paired (permutation-based) *t* tests^16^. Comparing MS ratio between the groups showed that both persistence ratio and prevalence ratio (computed as MS2/MS1) are significantly higher in SZ (*p*-values < 1e−4; Cohen’s *d* = 0.86 and 0.85).

In short, although immediate IPS transition probabilities seem to be minimally changed in SZ (Figure 5C), deep temporal organization of the states has been gravely altered in the patient group. Notably, this altered balance is such that patients spend more time in a poorly connected MS (i.e., MS2), which promotes phase decoupling within and across most functional networks – and anticoupling within the cognitive control network. Overall, this metastate analysis suggests that large-scale functional disconnection in SZ could be mediated by distortions in the deep temporal structure of IPS connectivity modes, at the network level.

This concludes our cluster-based analysis. In the next section, we will see the results of our cluster-free approach to IPS assessment, using the trajectory of instantaneous phase couplings. We will inspect whether phase synchrony evolution has been modulated in SZ.

### Phase Coupling Trajectory Is Less Efficient and Less Smooth in the Patient Group

In this section, we report the results of IPS trajectory characterization. We mentioned that collapsing IPS patterns onto one dimension (i.e., state labels) is a simplification that overlooks potentially useful information in the sample-to-sample connectomic changes (Miller et al., 2016). Hence, we used L1 distance to assess dissimilarity between time-indexed IPS matrices, and defined a number of measures to characterize the hyperspace and pathway traversed during each session. These measures are summarized in Table 2, and their empirical values are presented in Table 3.

**TABLE 3:** Trajectory analysis results.

**Measure**	**HC mean**	**SZ mean**	***p*-value (uncorrected)**	***p*-value (FDR corrected)**	**Effect size (Cohen’s *d*)**
Trajectory Length	3.93e+3	3.98e+3	0.096	0.096	−0.31
Span	63.25	61.94	0.0092	0.023	+0.50
Capacity	48.90	48.69	0.0004	0.002	+0.67
Efficiency	0.0125	0.0122	0.025	0.032	+0.36
Smoothness	3.35e−3	3.29e−3	0.0145	0.024	+0.46

*The measures were defined in Table 2.*

The results show that the average *trajectory length* is higher in SZ (compared to HC), but the difference is weakly significant (*p* = 0.096). However, all the other indices have significantly higher means in the HC group. Namely, the *span*, *capacity*, *efficiency*, and *smoothness* of the trajectory are all higher in normal subjects (FDR corrected *p*-values = 0.023, 0.002, 0.032, and 0.024, respectively). These results speak to the modulation of ongoing phase coupling in SZ, at the network level. This modulation is such that consecutive patterns are more dissimilar in the patient brain (hence the reduced smoothness), but this jumpy trajectory fails to achieve the more diverse repertoire of IPS patterns that is realized by the typical healthy brain.

**FIGURE 8:**
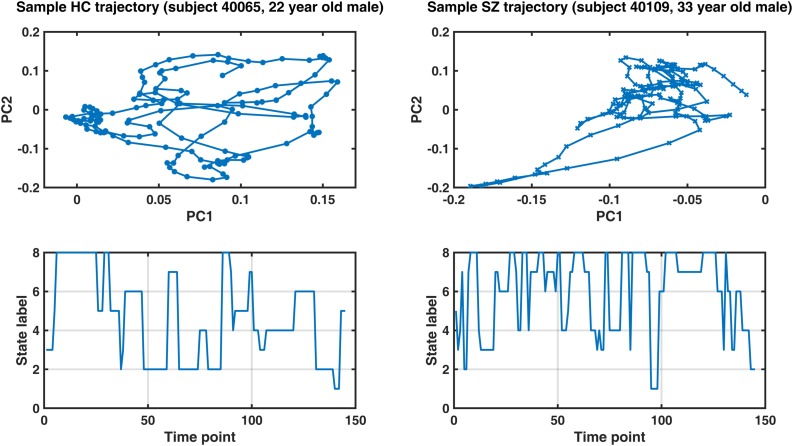
Sample IPS trajectories from a healthy control subject (HC, left panel) and a schizophrenia patient (SZ, right panel). Top panels: IPS trajectories in low-dimensional manifolds (spanned by the first two principal components, PC). Notably, the HC subject covers a larger space and realizes a smoother trajectory (compared to the patient). Lower panels illustrate the corresponding state sequences. The healthy subject spends longer periods in states 2 and 4 (which belong to metastate 1), while the patient’s sequence mostly avoids states 1 and 2, but visits states 5–8 more often (which are associated with metastate 2). Please refer to Table 3 for group differences in IPS trajectory measures.

Figure 8 shows representative IPS trajectories from a normal subject (22-year-old male) and a SZ patient (33-year-old male), projected on the first two principal components of their IPS profiles. Hence, each circle (or cross) is a time-indexed IPS pattern presented on a low-dimensional manifold and the connecting lines show the progression of IPS from time to time. Even visual inspection confirms that the HC trajectory smoothly explores a larger space (left panel) as opposed to the patient trajectory (right panel) which seems to be confined in the top right corner of the space most of the time. The corresponding state sequences (lower panels) show that the normal subject spends longer periods in state 2 and 4 (which belong to MS1). Conversely, the patient sequence mostly avoids states 1 and 2, but visits states 5–8 more often (which are associated with MS2).

Equipped with IPS measures derived from our state, metastate, and trajectory analyses, we will now look into the explanatory power of these phase indices for predicting SZ in a regression model.

### IPS Measures Predict SZ

**FIGURE 9:**
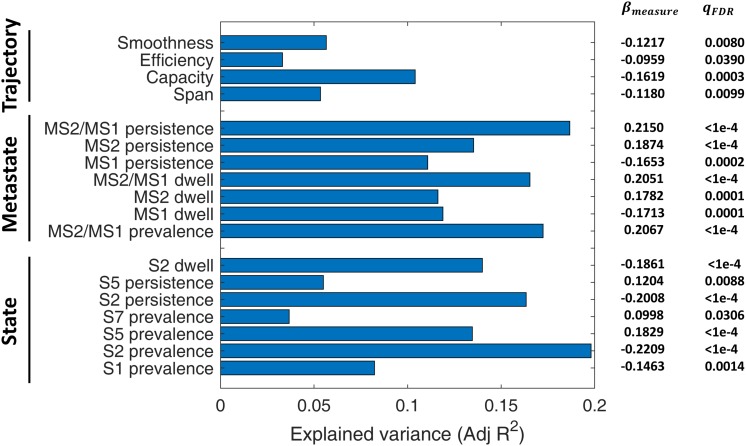
Diagnostic value of IPS measures for schizophrenia (SZ). A linear regression model (Eq. 7) was set up for each measure, while treating age, gender, and mean framewise displacement (MFD) as confounds. Bars denote adjusted *R*^2^ values (i.e., explained variances). Numbers on the right show regression coefficients (β_*measure*_) and FDR-corrected *p*-values (*q*_FDR_) of *F* tests conducted on regression models. The measures have been grouped into three categories: state-related measures, metastate-based measures, and trajectory measures. For definition of the measures, please refer to Table 2. S: State; MS: Metastate.

A linear regression model was used to assess the predictive power of different IPS measures for SZ diagnosis, while treating age, gender, and MFD as confounds. The adjusted ^*R^2*^ values (i.e., explained variances) have been reported in Figure 9, alongside the regression coefficients and FDR corrected *p*-values (of the *F* tests).

Among the included states, state 2 features (namely, its prevalence, persistence, and dwell) are the better predictors (adjusted *R*^2^ = 0.10). Notably, all the MS indices (i.e., MS prevalence, persistence, dwell, and MS2/MS1 ratio) have adjusted *R*^2^ above 0.12, while MS2/MS1 persistence ratio alone can explain 19% of the variance in diagnosis label. As such, metastates seem to be distinctive features. In fact, previous correlation-based DFC research has shown that metastate profile is heritable, subject-specific, and related to behavioral traits (Vidaurre et al., 2017). Among the trajectory measures, capacity seems the most distinctive, with adjusted *R*^2^ = 0.10. These results are particularly useful for selecting IPS features for classification studies.

### IPS States Are Distinct From Random Phase Synchrony Patterns

**FIGURE 10:**
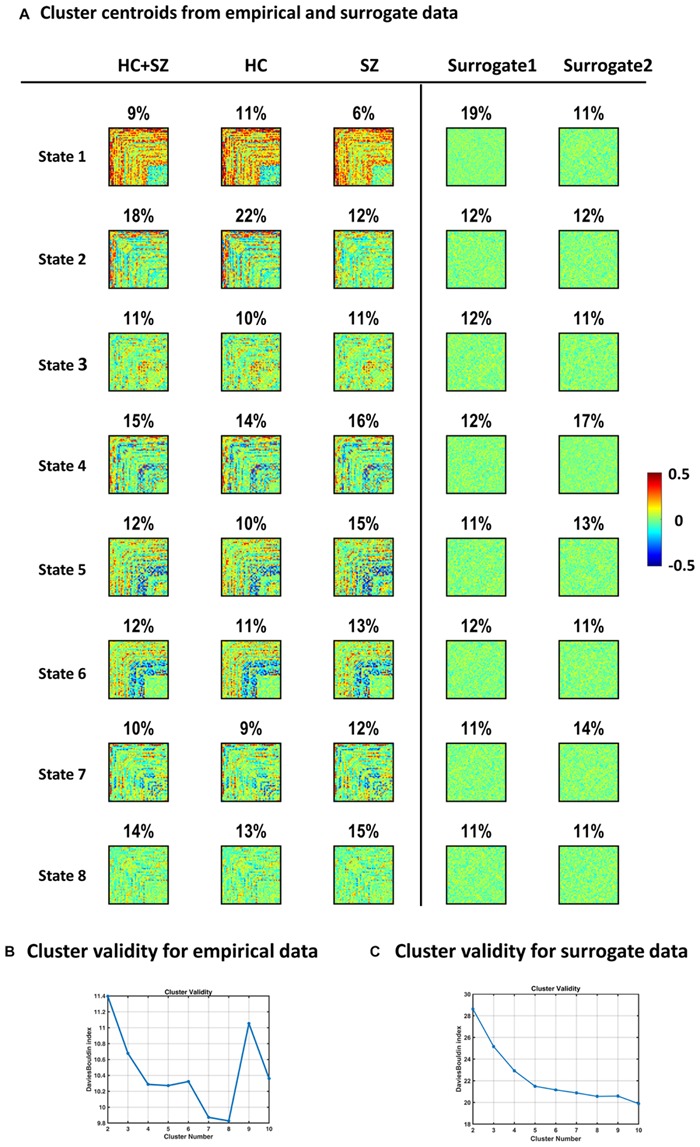
Empirical versus surrogate IPS states. Panel **(A)** plots show that clustering IPS patterns from surrogate data do not result in the same well-defined and distinct states identified in empirical data. The first column (left) shows the empirical states (same as Figure 4A). The second and third columns reflect the high correspondence of IPS states in the two (HC and SZ) groups. Percentage values denote the prevalence of each IPS mode. Conversely, the patterns in the last two columns (on the far right) were derived from surrogate data. Surrogate1 was generated by phase shuffling the original series and surrogate2 was produced by inducing random circular shifts in the original time courses. Surrogate data were subjected to the same clustering procedure as empirical data. The cluster validity plot for surrogate data [in panel **(C)**] shows that the Davies–Bouldin validity index continues to decrease by increasing model complexity (i.e., the cluster number). This is in contrast to the trend observed in real data [panel **(B)** here, same as Figure 3C], which endorses an optimal model size of 8. The surrogate states depicted in panel **(A)** show the clustering solution for *k* = 8, for the sake of comparison with empirical modes. The resultant surrogate states are flat and lack the strong coupling and anticoupling structure in the original states. Healthy Control: HC; Schizophrenic: SZ.

Figure 10A shows IPS states derived from clustering surrogate data, alongside empirical states. Surrogate1 was generated by phase shuffling the original series and surrogate2 was produced by inducing random circular shifts in the original data. For surrogate data, the cluster validity index (i.e., Davies–Bouldin index, panel C) continues to decrease by increasing model complexity, as opposed to the empirical plot (in panel B) that endorses eight clusters. Subsequent inspection of surrogate centroids (assuming *k* = 8 clusters) revealed that the resultant states are indistinguishable and lack the strong (anti)coupling structure in the empirical arrangements. Furthermore, we depicted group-specific states to highlight the correspondence of IPS modes in the two groups, although SZ patterns (especially states 1 and 2) are somewhat fainter.

In short, we see that empirical IPS modes are well-defined, diverse, and distinct from random phase synchrony patterns. In other words, IPS states seem to reflect intrinsic order in the phase synchronization of functional networks, which further corroborates the idea that resting-state dynamics may be conceptualized as excursion though a bounded repertoire of metastable functional modes (Deco et al., 2011; Cabral et al., 2017).

### Phase Synchrony States Are Not Driven by Head Motion

Correlation analysis showed that FD does not meaningfully covary with the occurrence of any state: *r* = 0.0323, 0.0198, 0.0023, −0.0286, 0.008, −0.0190, 0.0029, and −0.0141, for states 1–8, respectively. Moreover, the effect of state label on mean FD was insignificant [repeated-measures ANOVA: *F* (7,693) = 1.12, *p* = 0.35]. Hence, none of the states is associated with head movement, and the evidence does not support difference in the associated motion of the states.

**FIGURE 11:**
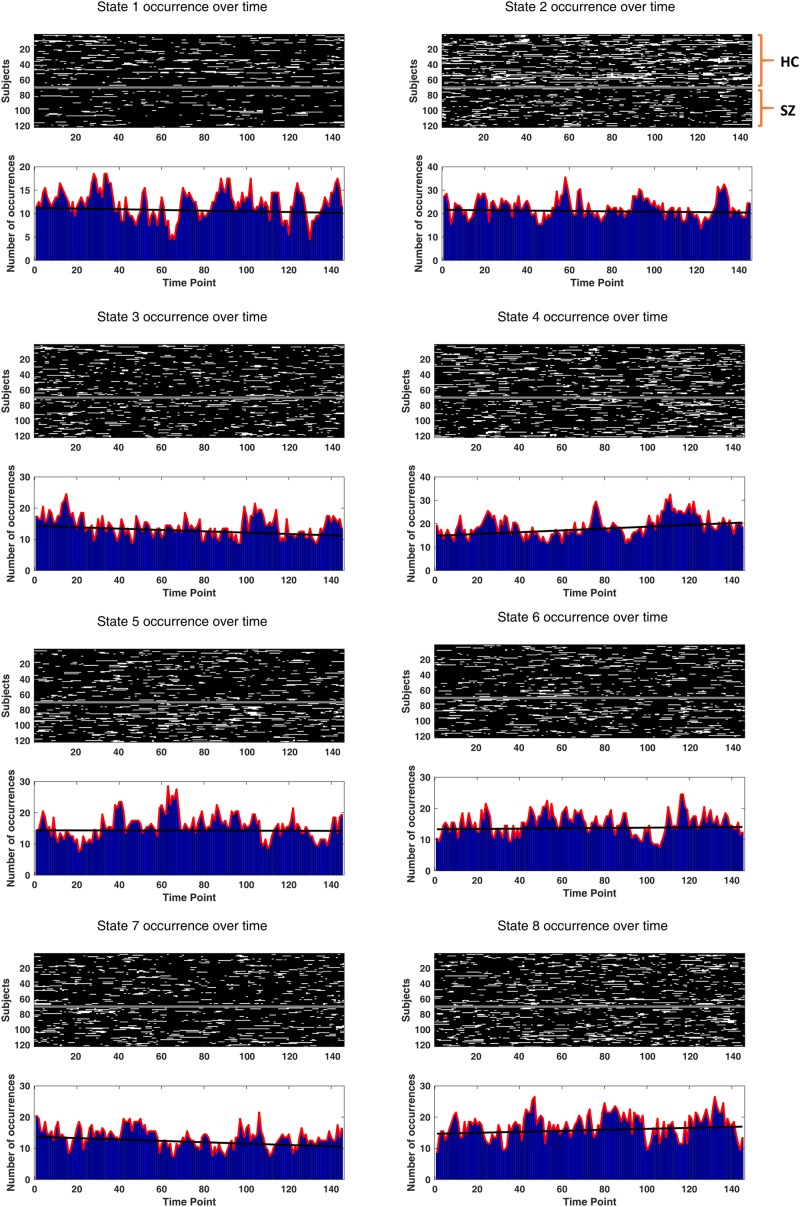
Drowsiness effect. The occurrence of each state (over time) has been depicted for each subject. The two groups (HC and SZ) have been separated by a constant (white) line, in the surface plots. Other white patches indicate the emergence of that particular state over time. In the lower bar plots, state occurrences have been accumulated over subjects, for each time point. The black fitted lines show group trends. Group trend for state 4 reveals a 3.9% increase in the expression of this state, per time sample. Only state 4 has a significantly non-zero (positive) trend (*p*-value = 0.034).

To verify the potential relation of the states to wakefulness, we inspected state occurrence rates over time. It turned out that only the best fitted line to state 4 incidence rate has a significantly non-zero (positive) trend. The uncorrected *p*-values (of one-sample *t* tests on the line slopes, across subjects) were 0.66, 0.69, 0.13, 0.034, 0.91, 0.76, 0.12, and 0.35, for the eight states, respectively. The actual state occurrences have been depicted in Figure 11, for all subjects (in the upper surface plots). In each lower plot, state occurrence has been accumulated over subjects per time point; hence, the superimposed fitted lines show group trends. Specifically, the group trend for state 4 reflects 3.9% increase in the appearance of this state, per time sample. Notably, state 4 was not different in prevalence or persistence between HC and SZ subjects (see Figures 5A,B).

## Discussion

Today, resting-state DFC features are being rigorously examined as potential functional biomarkers for diagnosis and prognosis of brain disorders. The ease of task-free data acquisition (especially from patients) makes resting-state studies particularly appealing. Besides, recent evidence suggests that resting-state DFC can affect task performance and may explain inter-subject differences in perception, learning, and other cognitive abilities (Gonzalez-castillo and Bandettini, 2019). Among DFC measures, fMRI IPS has the advantage of evading the window problem (Hindriks et al., 2015; Leonardi and Ville, 2015; Zalesky and Breakspear, 2015) and its functional relevance has been revealed in health, disease, and pharmacological conditions (Glerean et al., 2012; Ponce-Alvarez et al., 2015; Cabral et al., 2017; Alderson et al., 2019; Lord et al., 2019; Zhang et al., 2019). In the present study, we extended IPS characterization to higher functional and temporal scales, and addressed an important analysis issue – that of finding natural order in this type of data. In the following, we will review the key contributions of this work and the significance of the findings.

We showed that the inherent spatiotemporal order in IPS data can be uncovered (using spectral clustering) without compromising the phase coupling details. Importantly, this approach improves interpretability of the identified states. For instance, state 5 (which is more prevalent in SZ patients) shows clear anticoupling between CC and CB networks (see Figure 4). In fact, fronto-cerebellar dissociation in SZ has been commonly reported and relates to negative and cognitive symptoms, as well as executive dysfunction in SZ (Ridler et al., 2006; Brady et al., 2019). Moreover, cluster analysis revealed a globally coherent state (i.e., state 1), which has been reported in prior IPS research as well. Specifically, Cabral et al. (2017) showed that this highly synchronous state is visited less often in older adults with poor cognitive scores. Similarly, Lord et al. (2019) showed that the occurrence of a very synchronous state decreases after psilocybin injection. We found that state 1 is significantly less frequent in the SZ group, alongside state 2. As for state 2, it reflects strong interactions of SC and AUD with other networks. Functional (and structural) alterations of the subcortical network in SZ and their relationship with social and cognitive performance have been extensively studied in the literature (Koshiyama et al., 2018). It has even been suggested that reduced fronto-subcortical FC could be a functional biomarker of SZ (Vandevelde et al., 2017). As such, preserving the details of IPS patterns is quite informative and can reveal useful functional features (e.g., for diagnostic purposes).

Another important aspect of our research was focusing on IPS analysis at the network level (rather than regional), with a whole-brain scope, which speaks to higher-order functional integration in the brain. Notably, transient interaction of large-scale networks is a functional attribute that relates to personality traits (Vidaurre et al., 2017, 2019) and shows alterations in prior DFC accounts of SZ (Damaraju et al., 2014; Rashid et al., 2014; Salman et al., 2019). We used constrained ICA and verified (section “Subject-Specificity of Networks”) that this approach respects subject specificity of the estimated networks and simultaneously maintains network correspondence at the group level. Moreover, the fine-grained functional parcellation (with 50 ICs) was useful for inspection of sub-network behavior. For instance, we notice that four states reflect anticoupling among CC sub-components (i.e., states 4–7). Remarkably, three of these states belong to metastate 2, which is significantly predominant in SZ (Figure 7). This phase coupling alteration in the CC network of the patients might be related to cognitive deficits in SZ and supports previous reports of dysfunctional CC connections (Lesh et al., 2010; Alústiza et al., 2017). Hence, including sub-components of the networks is valuable in that it provides more insight into the functional organization of the states.

A further product of our analysis was uncovering the relationship between transition probability and IPS state similarity. Specifically, we showed that similar states (in terms of functional pattern and temporal profile) are more likely to switch into each other (Figure 5D). This is the same phenomenon reported in Vidaurre et al. (2017) – about gradual connectivity progression at rest – despite the fact that our connectivity states reflect phase synchrony modes and their states reflect correlations. Hence, the tendency to explore local neighborhoods in the (functional and temporal) feature space of metastable connectivity structures seems to be a fundamental aspect of spatiotemporal reorganization of the brain, at rest^17^.

Subsequently, we looked for deeper (slower) temporal order in the IPS transience. This analysis disclosed higher-level temporal organization of phase synchrony modes and the utility of metastate analysis. This hierarchical temporal arrangement seems to be another fundamental DFC trait that does not depend on the (correlation or phase based) nature of the connectivity states (Vidaurre et al., 2017). Moreover, metastate analysis turned out particularly useful in our clinical application. That is because patients and HCs do not differ much in their immediate transition profiles (Figure 5C); however, we noticed that MS balance has been gravely altered in SZ (Figure 7). In other words, it is the slower clock (which determines metastate occupation) that seems to be damaged in the SZ disorder. Importantly, metastate proportion turned out to be a strong predictor of SZ, in the regression analysis (Figure 9). Additionally, MS characterization showed that MS2 (which prevails in SZ) is significantly less coherent than MS1 (Figures 6B,C). Overall, we showed that SZ connectivity disorder is manifest on higher functional (i.e., network) and temporal (i.e., metastate) levels as well.

After state and metastate characterization, we focused on the trajectory of IPS evolution, free from the restrictions of cluster analysis. We defined novel measures (Table 2) to quantify different trajectory attributes. The results (in Table 3) showed that, despite the relatively large sample-to-sample jumps in SZ, the inefficient IPS trajectory of the patient brain precludes realization of a rich repertoire of FC patterns, compared to HC subjects. Hence, all four measures of span, capacity, efficiency, and smoothness were significantly lower in the patient group. Relevant research (Miller et al., 2016) has characterized the trajectory of (correlation-based) DFC patterns in SZ and showed that patient trajectories cover a smaller portion of the state space and realize fewer distinct patterns. These results seem also in line with the findings in Alderson et al. (2019), which indicate that cognitive performance is directly related to the *variety* of network configurations explored at rest. This makes more sense when we remember that cognitive deficits are among the core symptoms of SZ (Goldman-Rakic, 1994; Perlstein et al., 2001; Silver et al., 2003; Barch, 2005; Forbes et al., 2009).

Despite partial consistency of our findings with those in Miller et al. (2016) – about the reduced span of DFC space in SZ – IPS analysis shows that the connectivity trajectory length is actually longer in SZ, unlike the correlation-based result in Miller et al. (2016). A key difference is that we have followed the connectomic path in a higher dimensional space, and that IPS has inherently higher temporal resolution. This longer trajectory in SZ, alongside shorter span, results in the lower efficiency of FC evolution (Table 3), suggesting less structured navigation of the state space in SZ. Moreover, the recurrent inter-network IPS modes identified by our analysis do not resemble the complex-valued states (based on wavelet coherence) in Yaesoubi et al. (2015, 2017) or the correlation-based states in Allen et al. (2014), even though the same network parcellations were adopted in our studies^18^. This speaks to a well-defined identity for the IPS repertoire, beyond correlation-based^19^ DFC states. This disparity is due to the different mathematical properties of IPS and sliding-window correlations (Pedersen et al., 2018).

Lastly, we investigated the diagnostic value of IPS measures, using regression analysis. The metastates turned out to be distinctive traits for SZ identification, together with the prevalence of state 2 and the capacity of the trajectory. There is hope that, developing neuroimaging-based biomarkers and (machine learning based) classifiers would facilitate more objective diagnosis of patient (sub)groups, to furnish more effective treatment selection and prognosis (Drysdale et al., 2017; Fernandes et al., 2017; Bzdok and Meyer-Lindenberg, 2018). Along this way, FC-based methods can provide useful (statistical) insight into the pathological alterations of brain connectivity, at the observation level; however, characterizing the underlying neuronal circuitry and revealing the mechanisms of functional integration would call for model-based (effective connectivity) approaches (Friston, 2011, 2016; Gilson et al., 2019), which are recently being integrated with machine learning techniques as well (Frässle et al., 2020).

To elucidate the long-term vision, we mention a couple of recent achievements. Lately, Brady et al. (2019) have used a data-driven approach to show that connectivity breakdown between the cerebellum (CB) and right dorsolateral prefrontal cortex (DLPFC) directly corresponds to the severity of negative symptoms in SZ, which are known to be resistant to medication. Notably, when these researchers applied transcranial magnetic stimulation (TMS) to the cerebellar midline of SZ patients and restored this specific CB-DLPFC functional connection, the negative symptoms were also relieved – reflecting a causal relationship that is useful for therapeutic purposes. In another prominent research, Deco et al. (2019) have proposed a generative whole-brain model that can predict (*in silico*) how direct electrical stimulation of different brain regions would change the *proportion* of IPS states in the brain (of the sort depicted in our prevalence plot, in Figure 5A). As a proof of concept, the authors showed that this method can be used to “awaken” the brain from deep sleep to wakefulness and vice versa, i.e., switching between two conditions that entail different state proportions. Accordingly, 1 day we may be able to modulate disease-specific circuitry and restore state (or metastate) balance in brain disorders – such as SZ – which might relieve the clinical symptoms and eventually improve the quality of life for these patients.

## Data Availability Statement

The data analyzed in this study was obtained from the COllaborative Informatics and Neuroimaging Suite Data Exchange tool (COINS; https://coins.trendscenter.org/). Data collection was performed at the Mind Research Network, and funded by a Center of Biomedical Research Excellence (COBRE) grant 5P20RR021938/P20GM103472 from the NIH to Dr. Vince Calhoun.

## Author Contributions

TZ and G-AH-Z conceived the project. TZ analyzed the data and wrote the manuscript. G-AH-Z and FB discussed the results with TZ and reviewed and revised the manuscript. All authors approved this submission.

## Conflict of Interest

The authors declare that the research was conducted in the absence of any commercial or financial relationships that could be construed as a potential conflict of interest.

## Footnotes

1 Such as sliding window correlation and wavelet coherence analyses, which are not based solely on instantaneous phase relationships.

2 Yaesoubi et al. (2015, 2017) have accounted for IPS in identification of complex-valued recurrent functional modes using wavelet coherence, which contains both amplitude and phase coupling information. For more details, please refer to the “Discussion” section.

3 Schizophrenia symptoms include delusions, hallucinations, disorganized thinking and speech, cognitive dysfunction, flat expressions, and asociality (American Psychogeriatric Association, 2000).

4 The very term coined by the Swiss psychiatrist Eugen Bleuler (1857–1939) tends to signify a sort of “split” (schizen) in the normally integrated processes of the “mind” (phrenia) (Van Den Heuvel and Fornito, 2014).

5 The modulated connection are mostly between the prefrontal, temporal, and parietal cortices, as well as the striatum, thalamus, amygdala, and cerebellum (Nejad et al., 2012).

6 https://www.fil.ion.ucl.ac.uk/spm/software/spm12/

7 csICA improves spatial and temporal accuracy, subject specificity, statistical independence, and group correspondence of the recovered networks (Lin et al., 2010; Du and Fan, 2013; Salman et al., 2019).

8 Spatial ICA with high model order can achieve a detailed functional parcellation of cortical and subcortical structures with known anatomical and functional identity (Kiviniemi et al., 2009; Smith et al., 2009; Abou-Elseoud et al., 2010; Allen et al., 2014). However, model orders >100 have been shown to decrease ICA repeatability, as evaluated by ICASSO’s cluster quality index (Himberg et al., 2004; Abou-Elseoud et al., 2010).

9 http://trendscenter.org/software/gift/

10 An unsigned graph is more straightforward to manipulate and interpret algebraically than a signed graph (Gallier, 2016).

11 Laplacian matrix is defined as L = D − A, where A is the adjacency matrix and D is a diagonal matrix bearing the node degrees (Chung and Graham, 1997).

12 Shorthand for the eigenvectors corresponding to the *k* smallest eigenvalues.

13 The Davies–Bouldin criterion is based on the ratio of within-cluster scatter to between-cluster distance. Hence, the optimal clustering solution would achieve the lowest value of this index.

14 $L_{i\,j}^{m}=I\,P\,S^{m}\,\left(t_{j}\right)-I\,P\,S^{m}\,{\left(t_{i}\right)}_{1}$, where *m* is the subject index; *i*,*j* ∈ {1:*N*} and *N* is the number of time points per subject session.

15 In case of unequal session lengths (i.e., different number of time samples per subject), this measure can be normalized to the respective number of time points, to denote average step length.

16 FDR-corrected *p*-values for test on prevalence: *p* < 1e−4 (HC), *p* = 0.0165 (SZ); test on persistence: *p* < 1e−4 (HC), *p* = 0.0135 (SZ).

17 Note that if this gradual connectivity evolution were simply due to the continuous nature of fMRI signals or ICA time series, then the activity maps (associated with connectivity states) could have more readily reflected this gradual change – but they do not (Figure 5D, upper right panel). Moreover, the tendency of temporally similar connectivity states (in terms of prevalence and persistence profile) to follow each other in time cannot be explained by the continuity of fMRI/ICA time series (Figure 5D, lower panels).

18 Once collapsed along the frequency dimension, Yeasoubi’s FC states are almost identical to those in Allen et al. (2014), as depicted in Figure 5 of Yaesoubi et al. (2015), and both are remarkably different from IPS states. This comparison is licensed because our adopted functional networks are identical to theirs.

19 Coherence is also correlation in the frequency domain.

## References

[B1] Abou-Elseoud A. Starck T. Remes J. Nikkinen J. Tervonen O. Kiviniemi V. (2010). The effect of model order selection in group PICA. *Hum. Brain Mapp.* 31 1207–1216. 10.1002/hbm.20929 20063361 PMC6871136

[B2] Aggarwal C. C. Hinneburg A. Keim D. A. (2001). “On the surprising behavior of distance metrics in high dimensional space,” in *Database Theory — ICDT 2001. ICDT 2001. Lecture Notes in Computer Science*, Vol. 1973 eds Van den Bussche J. Vianu V., (Berlin: Springer), 420–434. 10.1007/3-540-44503-x_27

[B3] Alderson T. H. Bokde A. L. W. Kelso J. A. S. Maguire L. Coyle D. (2019). Metastable neural dynamics underlies cognitive performance across multiple behavioural paradigms. *bioRxiv* [Preprint] 10.1002/hbm.25009 PMC7375112 32301561

[B4] Allen E. A. Damaraju E. Plis S. M. Erhardt E. B. Eichele T. Calhoun V. D. (2014). Tracking whole-brain connectivity dynamics in the resting state. *Cereb. Cortex* 24 663–676. 10.1093/cercor/bhs352 23146964 PMC3920766

[B5] Allen E. A. Erhardt E. B. Damaraju E. Gruner W. Segall J. M. Silva R. F. (2011). A baseline for the multivariate comparison of resting-state networks. *Front. Syst. Neurosci.* 5:2. 10.3389/fnsys.2011.00002 21442040 PMC3051178

[B6] Alústiza I. Radua J. Pla M. Martin R. Ortuño F. (2017). Meta-analysis of functional magnetic resonance imaging studies of timing and cognitive control in schizophrenia and bipolar disorder : evidence of a primary time de fi cit. *Schizophr. Res.* 188 21–32. 10.1016/j.schres.2017.01.039 28169089

[B7] American Psychogeriatric Association, (2000). *DSM-IV-TR: Diagnostic and Statistical Manual of Mental Disorders, Text Revision*, Vol. 75 Washington, DC: American Psychogeriatric Association, 943 10.1002/jps.3080051129

[B8] Barch D. M. (2005). The cognitive neuroscience of schizophrenia. *Annu. Rev. Clin. Psychol.* 1 321–353. 10.1146/annurev.clinpsy.1.102803.143959 17716091

[B9] Beckmann C. Mackay C. Filippini N. Smith S. (2009). Group comparison of resting-state FMRI data using multi-subject ICA and dual regression. *Neuroimage* 47:S148 10.1016/s1053-8119(09)71511-3

[B10] Bedrosian E. (1962). A product theorem for Hilbert transforms. *Proc. IEEE* 51 868–869. 10.1109/PROC.1986.13495

[B11] Benjamini Y. Hochberg Y. (1995). Controlling the false discovery rate: a practical and powerful approach to multiple testing. *J. R. Stat. Soc. Ser. B* 57 289–300. 10.1111/j.2517-6161.1995.tb02031.x

[B12] Boashash B. (1992). Estimating and interpreting the instantaneous frequency of a signal-Part1: Fundamentals. *Proc. IEEE* 80 520–538. 10.1109/5.135376

[B13] Bonacich P. (2007). Some unique properties of eigenvector centrality. *Soc. Netw.* 29 555–564. 10.1016/j.socnet.2007.04.002

[B14] Bonacich P. Lloyd P. (2004). Calculating status with negative relations. *Soc. Netw.* 26 331–338. 10.1016/j.socnet.2004.08.007

[B15] Brady R. O. Gonsalvez I. Lee I. Öngür D. Seidman L. J. Schmahmann J. D. (2019). Cerebellar-prefrontal network connectivity and negative symptoms in schizophrenia. *Am. J. Psychiatry* 176 512–520. 10.1176/appi.ajp.2018.18040429 30696271 PMC6760327

[B16] Bzdok D. Meyer-Lindenberg A. (2018). Machine learning for precision psychiatry: opportunities and challenges. *Biol. Psychiatry Cogn. Neurosci. Neuroimaging* 3 223–230. 10.1016/j.bpsc.2017.11.007 29486863

[B17] Cabral J. Kringelbach M. L. Deco G. (2014). Exploring the network dynamics underlying brain activity during rest. *Prog. Neurobiol.* 114 102–131. 10.1016/j.pneurobio.2013.12.005 24389385

[B18] Cabral J. Vidaurre D. Marques P. Magalhães R. Silva Moreira P. Miguel Soares J. (2017). Cognitive performance in healthy older adults relates to spontaneous switching between states of functional connectivity during rest. *Sci. Rep.* 7 1–13. 10.1038/s41598-017-05425-7 28698644 PMC5506029

[B19] Calhoun V. D. Adali T. Pearlson G. D. Pekar J. J. (2001). A method for making group inferences from functional MRI data using independent component analysis. *Hum. Brain Mapp.* 14 140–151. 10.1002/hbm.1048 11559959 PMC6871952

[B20] Calhoun V. D. Miller R. Pearlson G. Adali T. Adalı T. Adal T. (2014). The chronnectome: time-varying connectivity networks as the next frontier in fMRI data discovery. *Neuron* 84 262–274. 10.1016/j.neuron.2014.10.015 25374354 PMC4372723

[B21] Çetin M. S. Christensen F. Abbott C. C. Stephen J. M. Mayer A. R. Cañive J. M. (2014). Thalamus and posterior temporal lobe show greater inter-network connectivity at rest and across sensory paradigms in schizophrenia. *Neuroimage* 97 117–126. 10.1016/j.neuroimage.2014.04.009 24736181 PMC4087193

[B22] Chang C. Glover G. H. (2010). Time–frequency dynamics of resting-state brain connectivity measured with fMRI. *Neuroimage* 50 81–98. 10.1016/j.neuroimage.2009.12.011 20006716 PMC2827259

[B23] Chen Z. Calhoun V. (2018). Effect of spatial smoothing on task fMRI ICA and functional connectivity. *Front. Neurosci.* 12:15. 10.3389/fnins.2018.00015 29456485 PMC5801305

[B24] Chung F. R. K. Graham F. C. (1997). *Spectral Graph Theory.* Providence, RI: American Mathematical Soc.

[B25] Collins D. L. Zijdenbos A. P. Kollokian V. Sied J. G. Kabani N. J. Holmes C. J. (1998). Design and construction of a realistic digital brain phantom. *IEEE Trans. Med. Imaging* 17 463–468. 10.1109/42.712135 9735909

[B26] Cordes D. Haughton V. M. Arfanakis K. Carew J. D. Turski P. A. Moritz C. H. (2001). Frequencies contributing to functional connectivity in the cerebral cortex in “resting-state” data. *Am. J. Neuroradiol.* 22 1326–1333. 11498421 PMC7975218

[B27] Damaraju E. Allen E. A. Belger A. Ford J. M. McEwen S. Mathalon D. H. (2014). Dynamic functional connectivity analysis reveals transient states of dysconnectivity in schizophrenia. *NeuroImage Clin.* 5 298–308. 10.1016/j.nicl.2014.07.003 25161896 PMC4141977

[B28] Davies D. L. Bouldin D. W. (1979). A cluster separation measure. *IEEE Trans. Pattern Anal. Mach. Intell.* 1 224–227. 10.1109/TPAMI.1979.4766909 21868852

[B29] de Lacy N. Calhoun V. D. (2019). Dynamic connectivity and the effects of maturation in youth with attention deficit hyperactivity disorder. *Netw. Neurosci.* 3 195–216. 10.1162/netn_a_00063 30793080 PMC6372020

[B30] Deco G. Cruzat J. Cabral J. Tagliazucchi E. Laufs H. Logothetis N. K. (2019). Awakening: predicting external stimulation to force transitions between different brain states. *Proc. Natl. Acad. Sci. U.S.A.* 116 18088–18097. 10.1073/pnas.1905534116 31427539 PMC6731634

[B31] Deco G. Jirsa V. K. McIntosh A. R. (2011). Emerging concepts for the dynamical organization of resting-state activity in the brain. *Nat. Rev. Neurosci.* 12 43–56. 10.1038/nrn2961 21170073

[B32] Deco G. Jirsa V. K. McIntosh A. R. (2013). Resting brains never rest: computational insights into potential cognitive architectures. *Trends Neurosci.* 36 268–274. 10.1016/j.tins.2013.03.001 23561718

[B33] Deco G. Kringelbach M. L. Jirsa V. K. Ritter P. (2017). The dynamics of resting fluctuations in the brain: metastability and its dynamical cortical core. *Sci. Rep.* 7 1–14. 10.1038/s41598-017-03073-5 28596608 PMC5465179

[B34] Deshmukh A. V. Shivhare V. Gadre V. M. Patkar D. P. Shah S. Pungavkar S. (2004). “A phase based method for investigating the functional connectivity in the fMRI data,” in *Proceedings of the IEEE INDICON 2004 - 1st India Annual Conference*, Kharagpur, 272–277. 10.1109/INDICO.2004.1497754

[B35] Díez-cirarda M. Strafella A. P. Kim J. Peña J. Ojeda N. (2018). Dynamic functional connectivity in Parkinson’ s disease patients with mild cognitive impairment and normal cognition. *NeuroImage Clin.* 17 847–855. 10.1016/j.nicl.2017.12.013 29527489 PMC5842729

[B36] Drysdale A. T. Grosenick L. Downar J. Dunlop K. Mansouri F. Meng Y. (2017). Resting-state connectivity biomarkers define neurophysiological subtypes of depression. *Nat. Med.* 23 28–38. 10.1038/nm.4246 27918562 PMC5624035

[B37] Du Y. Fan Y. (2013). Group information guided ICA for fMRI data analysis. *Neuroimage* 69 157–197. 10.1016/j.neuroimage.2012.11.008 23194820

[B38] Du Y. Fryer S. L. Fu Z. Lin D. Sui J. Chen J. (2018). Dynamic functional connectivity impairments in early schizophrenia and clinical high-risk for psychosis. *Neuroimage* 180(Pt B) 632–645. 10.1016/j.neuroimage.2017.10.022 29038030 PMC5899692

[B39] Du Y. Fu Z. Sui J. Gao S. Xing Y. Lin D. (2019). NeuroMark : an adaptive independent component analysis framework for estimating reproducible and comparable fMRI biomarkers among brain disorders. *medRxiv* [Preprint]. 10.1101/19008631

[B40] Erhardt E. B. Rachakonda S. Bedrick E. J. Allen E. A. Adali T. Calhoun V. D. (2011). Comparison of multi-subject ICA methods for analysis of fMRI data. *Hum. Brain Mapp.* 32 2075–2095. 10.1002/hbm.21170 21162045 PMC3117074

[B41] Espinoza F. A. Anderson N. E. Vergara V. M. Harenski C. L. Decety J. Rachakonda S. (2019). Resting-state fMRI dynamic functional network connectivity and associations with psychopathy traits. *NeuroImage Clin.* 24:101970. 10.1016/j.nicl.2019.101970 31473543 PMC6728837

[B42] Fernandes B. S. Williams L. M. Steiner J. Leboyer M. Carvalho A. F. Berk M. (2017). The new field of ‘precision psychiatry’. *BMC Med.* 15:80. 10.1186/s12916-017-0849-x 28403846 PMC5390384

[B43] Fiedler M. (1973). Algebraic connectivity of graphs. *Czechoslov. Math. J.* 23 298–305.

[B44] First M. B. Spitzer R. L. Gibbon M. Williams J. B. (2002). *Structured Clinical Interview for DSM-IV-TR Axis I Disorders*. research version, patient edition (SCID-I/P). New York, NY: Biometrics Research, New York State Psychiatric Institute.

[B45] Forbes N. F. Carrick L. A. McIntosh A. M. Lawrie S. M. (2009). Working memory in schizophrenia: a meta-analysis. *Psychol. Med.* 36 889–905. 10.1017/S0033291708004558 18945379

[B46] Fornito A. Zalesky A. Pantelis C. Bullmore E. T. (2012). Schizophrenia, neuroimaging and connectomics. *Neuroimage* 62 2296–2314. 10.1016/j.neuroimage.2011.12.090 22387165

[B47] Fox M. D. Snyder A. Z. Vincent J. L. Corbetta M. Essen D. C. Van Raichle M. E. (2005). The human brain is intrinsically organized into dynamic, anticorrelated functional networks. *Proc. Natl. Acad. Sci. U.S.A.* 102 9673–9678. 10.1073/pnas.0504136102 15976020 PMC1157105

[B48] Frässle S. Marquand A. F. Schmaal L. Dinga R. Veltman D. J. van der Wee N. J. A. (2020). Predicting individual clinical trajectories of depression with generative embedding. *NeuroImage Clin.* 102213. (in press) 10.1016/j.nicl.2020.102213 PMC7082217 32197140

[B49] Friston K. J. (1998). The disconnection hypothesis. *Schizophr. Res.* 30 115–125. 10.1016/S0920-9964(97)00140-0 9549774

[B50] Friston K. J. (2011). Functional and effective connectivity: a Review. *Brain Connect.* 1 13–36. 10.1089/brain.2011.0008 22432952

[B51] Friston K. J. (2016). Computational nosology and precision psychiatry. *Comput. Psychiatry* 1 2–23. 10.7551/mitpress/9780262035422.003.0011 29400354 PMC5774181

[B52] Fu Z. Caprihan A. Chen J. Du Y. Adair J. C. Sui J. (2019). Altered static and dynamic functional network connectivity in Alzheimer’s disease and subcortical ischemic vascular disease: shared and specific brain connectivity abnormalities. *Hum. Brain Mapp.* 40 3203–3221. 10.1002/hbm.24591 30950567 PMC6865624

[B53] Gallier J. (2016). Spectral theory of unsigned and signed graphs. applications to graph clustering: a survey. *arXiv* [Preprint]; arXiv1601.04692

[B54] Ghosh A. Rho Y. McIntosh A. R. Kotter R. Jirsa V. K. (2008). Noise during rest enables the exploration of the brain’s dynamic repertoire. *PLoS Comput. Biol.* 4:e1000196. 10.1371/journal.pcbi.1000196 18846206 PMC2551736

[B55] Gilbert N. Bernier R. A. Calhoun V. D. Brenner E. Grossner E. Rajtmajer S. M. (2018). Diminished neural network dynamics after moderate and severe traumatic brain injury. *PLoS One* 13:e0197419. 10.1371/journal.pone.0197419 29883447 PMC5993261

[B56] Gilson M. Zamora-López G. Pallarés V. Adhikari M. H. Senden M. Tauste Campo A. (2019). Model-based whole-brain effective connectivity to study distributed cognition in health and disease. *Netw. Neurosci.* 1–62. (in press) 10.1162/netn_a_00117 32537531 PMC7286310

[B57] Glerean E. Salmi J. Lahnakoski J. M. Jääskeläinen I. P. Sams M. (2012). Functional magnetic resonance imaging phase synchronization as a measure of dynamic functional connectivity. *Brain Connect.* 2 91–101. 10.1089/brain.2011.0068 22559794 PMC3624768

[B58] Goldman-Rakic P. S. (1994). Working memory dysfunction in schizophrenia. *J. Neuropsychiatry Clin. Neurosci.* 6 348–357. 10.1176/jnp.6.4.348 7841806

[B59] Gonzalez-castillo J. Bandettini P. A. (2019). Task-based dynamic functional connectivity: recent findings and open questions. *Neuroimage* 180(Pt B) 526–533. 10.1016/j.neuroimage.2017.08.006 28780401 PMC5797523

[B60] Himberg J. Hyvärinen A. Esposito F. (2004). Validating the independent components of neuroimaging time series via clustering and visualization. *Neuroimage* 22 1214–1222. 10.1016/j.neuroimage.2004.03.027 15219593

[B61] Hindriks R. Adhikari M. H. Murayama Y. Ganzetti M. Mantini D. Logothetis N. K. (2015). Can sliding-window correlations reveal dynamic functional connectivity in resting-state fMRI? *Neuroimage* 127 242–256. 10.1016/j.neuroimage.2015.11.055 26631813 PMC4758830

[B62] Hutchison R. M. Womelsdorf T. Allen E. A. Bandettini P. A. Calhoun V. D. Corbetta M. (2013). Dynamic functional connectivity: promise, issues, and interpretations. *Neuroimage* 80 360–378. 10.1016/j.neuroimage.2013.05.079 23707587 PMC3807588

[B63] Keilholz S. D. (2014). The neural basis of time-varying resting-state functional connectivity. *Brain Connect.* 4 769–779. 10.1089/brain.2014.0250 24975024 PMC4268576

[B64] Keilholz S. D. Magnuson M. E. Pan W. J. Willis M. Thompson G. J. (2013). Dynamic properties of functional connectivity in the rodent. *Brain Connect.* 3 31–40. 10.1089/brain.2012.0115 23106103 PMC3621313

[B65] Kitzbichler M. G. Smith M. L. Christensen S. R. Bullmore E. (2009). Broadband criticality of human brain network synchronization. *PLoS Comput. Biol.* 5:e1000314. 10.1371/journal.pcbi.1000314 19300473 PMC2647739

[B66] Kiviniemi V. Starck T. Remes J. Long X. Nikkinen J. Haapea M. (2009). Functional segmentation of the brain cortex using high model order group PICA. *Hum. Brain Mapp.* 30 3865–3886. 10.1002/hbm.20813 19507160 PMC6870574

[B67] Koshiyama D. Fukunaga M. Okada N. Yamashita F. (2018). Role of subcortical structures on cognitive and social function in schizophrenia. *Sci. Rep.* 8:1183 10.1038/s41598-017-18950-2 PMC5775279 29352126

[B68] Lachaux J. P. Rodriguez E. Le Van Quyen M. Lutz A. Martinerie J. Varela F. J. (2000). Studying single-trials of phase synchronous activity in the brain. *Int. J. Bifurcat. Chaos*. 10 2429–2439. 10.1142/S0218127400001560

[B69] Lachaux J. P. Rodriguez E. Martinerie J. Varela F. J. (1999). Measuring phase synchrony in brain signals. *Hum. Brain Mapp.* 8 194–208. 10.1002/(SICI)1097-019319998:4<194::AID-HBM4>3.0.CO;2-C 10619414 PMC6873296

[B70] Laird A. R. Rogers B. P. Carew J. D. Arfanakis K. Moritz C. H. Meyerand M. E. (2002). Characterizing instantaneous phase relationships in whole-brain fMRI activation data. *Hum. Brain Mapp.* 16 71–80. 10.1002/hbm.10027 11954057 PMC6872093

[B71] Leonardi N. Ville D. Van De (2015). On spurious and real fluctuations of dynamic functional connectivity during rest. *Neuroimage* 104 430–436. 10.1016/j.neuroimage.2014.09.007 25234118

[B72] Lesh T. A. Niendam T. A. Minzenberg M. J. Carter C. S. (2010). Cognitive control deficits in schizophrenia : mechanisms and meaning. *Neuropsychopharmacology* 36 316–338. 10.1038/npp.2010.156 20844478 PMC3052853

[B73] Liang M. Zhou Y. Jiang T. Liu Z. Tian L. Liu H. (2006). Widespread functional disconnectivity in schizophrenia with resting-state functional magnetic resonance imaging. *Neuroreport* 17 209–213. 10.1097/01.wnr.0000198434.06518.b8 16407773

[B74] Lin Q. Liu J. Zheng Y. Liang H. Calhoun V. D. (2010). Semiblind spatial ICA of fMRI using spatial constraints. *Hum Brain Mapp.* 31 1076–1088. 10.1002/hbm.20919 20017117 PMC2891131

[B75] Lord L. Expert P. Atasoy S. Roseman L. Rapuano K. (2019). Dynamical exploration of the repertoire of brain networks at rest is modulated by psilocybin neuroimage dynamical exploration of the repertoire of brain networks at rest is modulated by psilocybin. *Neuroimage* 199 127–142. 10.1016/j.neuroimage.2019.05.060 31132450

[B76] Luxburg U. Von (2007). A tutorial on spectrum clustering. *Stat. Comput.* 17 1–32. 10.1055/s-0034-1376191 28373156 PMC5394262

[B77] Ma S. Calhoun V. D. Phlypo R. Adali T. (2014). Dynamic changes of spatial functional network connectivity in healthy individuals and schizophrenia patients using independent vector analysis. *Neuroimage* 90 196–206. 10.1016/j.neuroimage.2013.12.063 24418507 PMC5061129

[B78] Mcclellan J. H. Parks T. W. Rabiner L. R. (1973). A computer program for designing optimum FIR linear phase digital filters. *IEEE Trans. Audio Electroacoust.* 21 506–526. 10.1109/TAU.1973.1162525

[B79] Miller R. L. Yaesoubi M. Turner J. A. Mathalon D. Preda A. Pearlson G. (2016). Higher dimensional meta-state analysis reveals reduced resting fMRI connectivity dynamism in schizophrenia patients. *PLoS One* 11:e0149849. 10.1371/journal.pone.0149849 26981625 PMC4794213

[B80] Murphy K. Fox M. D. (2017). Towards a consensus regarding global signal regression for resting state functional connectivity MRI. *Neuroimage* 154 169–173. 10.1016/j.neuroimage.2016.11.052 27888059 PMC5489207

[B81] Murray C. J. L. Barber R. M. Foreman K. J. Ozgoren A. A. Abd-Allah F. Abera S. F. (2016). Global, regional, and national disability-adjusted life years (DALYs) for 306 diseases and injuries and healthy life expectancy (HALE) for 188 countries, 1990-2013: quantifying the epidemiological transition. *Lancet* 386 2145–2191. 10.1016/S0140-6736(15)61340-X 26321261 PMC4673910

[B82] Nejad A. B. Ebdrup B. H. Glenthøj B. Y. Siebner H. R. (2012). Brain connectivity studies in schizophrenia: unravelling the effects of antipsychotics. *Curr. Neuropharmacol.* 10 219–230. 10.2174/157015912803217305 23449679 PMC3468876

[B83] Ng A. Y. Jordan M. I. Weiss Y. (2002). “On spectral clustering: analysis and an algorithm,” *NIPS’01: Proceedings of the 14th International Conference on Neural Information Processing Systems: Natural and Synthetic*, 849–856.

[B84] Pedersen M. Omidvarnia A. Zalesky A. Jackson G. D. (2018). On the relationship between instantaneous phase synchrony and correlation-based sliding windows for time-resolved fMRI connectivity analysis. *Neuroimage* 181 85–94. 10.1016/j.neuroimage.2018.06.020 29890326

[B85] Perlstein W. M. Carter C. S. Noll D. C. Cohen J. D. (2001). Relation of prefrontal cortex dysfunction to working memory and symptoms in schizophrenia. *Am. J. Psychiatry*. 158 1105–1113. 10.1176/appi.ajp.158.7.1105 11431233

[B86] Politis D. N. Romano J. P. (1992). *A Circular Block**-Resampling Procedure for Stationary Data. ExploreLimits Bootstrap* 2635270 New York, NY: John Wiley & Sons.

[B87] Ponce-Alvarez A. Deco G. Hagmann P. Romani G. L. Mantini D. Corbetta M. (2015). Resting-state temporal synchronization networks emerge from connectivity topology and heterogeneity. *PLoS Comput. Biol.* 11:e1004100. 10.1371/journal.pcbi.1004100 25692996 PMC4333573

[B88] Power J. D. Barnes K. A. Snyder A. Z. Schlaggar B. L. Petersen S. E. (2012). Spurious but systematic correlations in functional connectivity MRI networks arise from subject motion. *Neuroimage*. 59 2142–2154. 10.1016/j.neuroimage.2011.10.018 22019881 PMC3254728

[B89] Preti M. G. Bolton T. A. Van De Ville D. (2017). The dynamic functional connectome: state-of-the-art and perspectives. *Neuroimage* 160 41–54. 10.1016/j.neuroimage.2016.12.061 28034766

[B90] Rabinovich M. I. Varona P. (2011). Robust transient dynamics and brain functions. *Front. Comput. Neurosci.* 5:24. 10.3389/fncom.2011.00024 21716642 PMC3116137

[B91] Rashid B. Blanken L. M. E. Muetzel R. L. Miller R. Damaraju E. Arbabshirani M. R. (2018). Connectivity dynamics in typical development and its relationship to autistic traits and autism spectrum disorder. *Hum. Brain Mapp.* 39 3127–3142. 10.1002/hbm.24064 29602272 PMC6045960

[B92] Rashid B. Damaraju E. Pearlson G. D. Calhoun V. D. (2014). Dynamic connectivity states estimated from resting fMRI Identify differences among Schizophrenia, bipolar disorder, and healthy control subjects. *Front. Hum. Neurosci.* 8:897. 10.3389/fnhum.2014.00897 25426048 PMC4224100

[B93] Ridler K. Veijola J. M. Tanskanen P. Miettunen J. Chitnis X. Suckling J. (2006). Fronto-cerebellar systems are associated with infant motor and adult executive functions in healthy adults but not in schizophrenia. *Proc. Natl. Acad. Sci. U.S.A.* 103 15651–15656. 10.1073/pnas.0602639103 17028177 PMC1636802

[B94] Riolo M. A. Newman M. E. J. (2014). First-principles multiway spectral partitioning of graphs. *J. Complex Netw.* 2 121–140. 10.1093/comnet/cnt021

[B95] Salman M. S. Du Y. Lin D. Fu Z. Fedorov A. Damaraju E. (2019). Group ICA for identifying biomarkers in schizophrenia : ‘Adaptive’ networks via spatially constrained ICA show more sensitivity to group di ff erences than spatio-temporal regression. *NeuroImage Clin.* 22:101747 10.1016/j.nicl.2019.101747 PMC6438914 30921608

[B96] Schreiber T. Schmitz A. (2000). Surrogate time series. *Phys. D Nonlinear Phenom.* 142 346–382. 10.1016/S0167-2789(00)00043-9

[B97] Shi J. Malik J. (2000). Normalized cuts and image segmentation part of the electrical and computer engineering commons recommended citation normalized cuts and image segmentation normalized cuts and image segmentation. *IEEE Trans. Pattern Anal. Mach. Intell.* 22 888–905. 10.1109/34.868688

[B98] Shirer W. R. Ryali S. Rykhlevskaia E. Menon V. Greicius M. D. (2012). Decoding subject-driven cognitive states with whole-brain connectivity patterns. *Cereb. Cortex* 22 158–165. 10.1093/cercor/bhr099 21616982 PMC3236795

[B99] Silver H. Feldman P. Bilker W. Gur R. C. (2003). Working memory deficit as a core neuropsychological dysfunction in schizophrenia. *Am. J. Psychiatry* 160 1809–1816. 10.1176/appi.ajp.160.10.1809 14514495

[B100] Smith S. M. Fox P. T. Miller K. L. Glahn D. C. Fox P. M. Mackay C. E. (2009). Correspondence of the brain’s functional architecture during activation and rest. *Proc. Natl. Acad. Sci. U.S.A.* 106 13040–13045. 10.1073/pnas.0905267106 19620724 PMC2722273

[B101] Sun J. Hong X. Tong S. Member S. (2012). phase synchronization analysis of EEG signals : an evaluation based on surrogate tests. *IEEE Trans. Biomed. Eng.* 59 2254–2263. 10.1109/TBME.2012.2199490 22665500

[B102] Tagliazucchi E. Laufs H. Garrett D. D. (2015). Multimodal imaging of dynamic functional connectivity. *Front. Neurol.* 6:10 10.3389/fneur.2015.00010 PMC4329798 25762977

[B103] Tagliazucchi E. von Wegner F. Morzelewski A. Brodbeck V. Laufs H. (2012). Dynamic BOLD functional connectivity in humans and its electrophysiological correlates. *Front. Hum. Neurosci.* 6:339. 10.3389/fnhum.2012.00339 23293596 PMC3531919

[B104] Tass P. Rosenblum M. G. Weule J. Kurths J. Pikovsky A. Volkmann J. (1998). Detection of n:m phase locking from noisy data: application to magnetoencephalography. *Phys. Rev. Lett.* 81 3291–3294. 10.1103/PhysRevLett.81.3291

[B105] Theiler J. Eubank S. Longtin A. Galdrikian B. Doyne Farmer J. (1992). Testing for nonlinearity in time series: the method of surrogate data. *Phys. D Nonlinear Phenom.* 58 77–94. 10.1016/0167-2789(92)90102-S

[B106] Thompson G. J. Pan W. J. Magnuson M. E. Jaeger D. Keilholz S. D. (2014). Quasi-periodic patterns (QPP): large-scale dynamics in resting state fMRI that correlate with local infraslow electrical activity. *Neuroimage* 84 1018–1031. 10.1016/j.neuroimage.2013.09.029 24071524 PMC3869452

[B107] Van Den Heuvel M. P. Fornito A. (2014). Brain networks in schizophrenia. *Neuropsychol. Rev.* 24 32–48. 10.1007/s11065-014-9248-7 24500505

[B108] Vandevelde A. Leroux E. Delcroix N. Dollfus S. (2017). Fronto-subcortical functional connectivity in patients with schizophrenia and bipolar disorder during a verbal fluency task. *World J. Biol. Psychiatry* 19(Suppl. 3) S124–S132. 10.1080/15622975.2017.1349339 28669318

[B109] Vidaurre A. D. Llera A. Smith S. M. Woolrich M. W. (2019). Behavioural relevance of spontaneous, transient brain network interactions in fMRI. *bioRxiv* [Preprint] 10.1016/j.neuroimage.2020.117713 PMC7994296 33421594

[B110] Vidaurre D. Smith S. M. Woolrich M. W. (2017). Brain network dynamics are hierarchically organized in time. *Proc. Natl. Acad. Sci. U.S.A.* 114 12827–12832. 10.1073/pnas.1705120114 29087305 PMC5715736

[B111] Yaesoubi M. Allen E. A. Miller R. L. Calhoun V. D. (2015). Dynamic coherence analysis of resting fMRI data to jointly capture state-based phase, frequency, and time-domain information. *Neuroimage* 120 133–142. 10.1016/j.neuroimage.2015.07.002 26162552 PMC4589498

[B112] Yaesoubi M. Miller R. L. Bustillo J. Lim K. O. Vaidya J. Calhoun V. D. (2017). A joint time-frequency analysis of resting-state functional connectivity reveals novel patterns of connectivity shared between or unique to schizophrenia patients and healthy controls. *NeuroImage Clin.* 15 761–768. 10.1016/j.nicl.2017.06.023 28706851 PMC5496209

[B113] Zalesky A. Breakspear M. (2015). Towards a statistical test for functional connectivity dynamics. *Neuroimage* 114 466–470. 10.1016/j.neuroimage.2015.03.047 25818688

[B114] Zhang R. Kranz G. S. Lee T. M. C. (2019). Functional connectome from phase synchrony at resting state is a neural fingerprint. *Brain Connect.* 9 519–528. 10.1089/brain.2018.0657 30997813

