# Supplementary material for: Deep Temporal Organization of fMRI Phase Synchrony Modes Promotes Large-Scale Disconnection in Schizophrenia
Source: Front Neurosci. 2020 Mar 27;14:214. doi: 10.3389/fnins.2020.00214 (PMC7118690; doi:10.3389/fnins.2020.00214)
Supplement: Supplementary file 1 [file Data_Sheet_1.pdf]

Supplementary Material

Deep temporal organization of fMRI phase synchrony modes promotes large-scale disconnection in schizophrenia

Tahereh S. Zarghami, Gholam-Ali Hossein-Zadeh, Fariba Bahrami

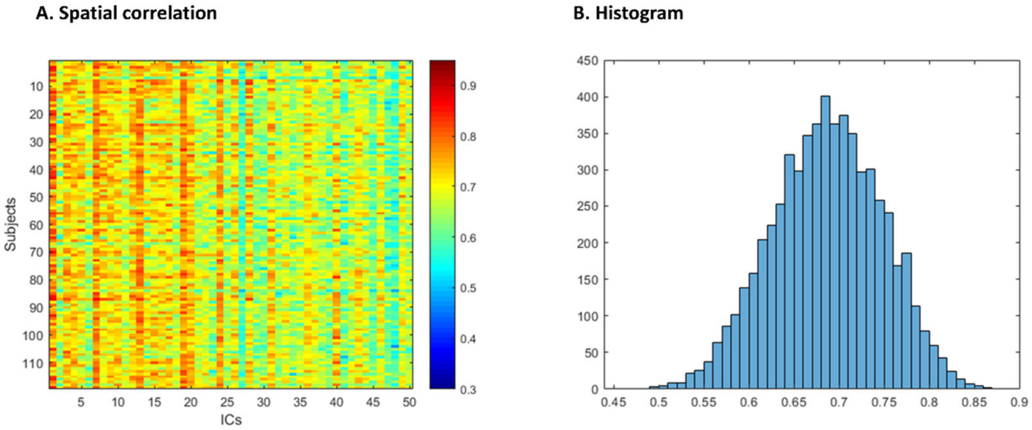

Figure S1: Subject-specificity of networks. (A) Normalized spatial correlation of subject-specific ICs with the 50 IC templates used to run constrained ICA. (B) Histogram of normalized spatial correlation values (for all subjects and ICs).

Table S1: Trajectory analysis results, for data preprocessed using Gaussian smoothing kernel with FWHM= 9 mm. The measures are defined in Table 2.

Measure	HC Mean	SZ Mean	P-value (Uncorrected)	P-value (FDR corrected)	Effect Size (Cohen's d)
Trajectory Length	6625.4	6757.9	0.0083	0.016	-0.45
Span	73.15	73.05	0.82	0.82	+0.04
Capacity	51.39	50.98	0.013	0.016	+0.48
Efficiency	0.0078	0.0075	0.0019	0.009	+0.50
Smoothness	3.41e-3	3.34e-3	0.012	0.016	+0.47

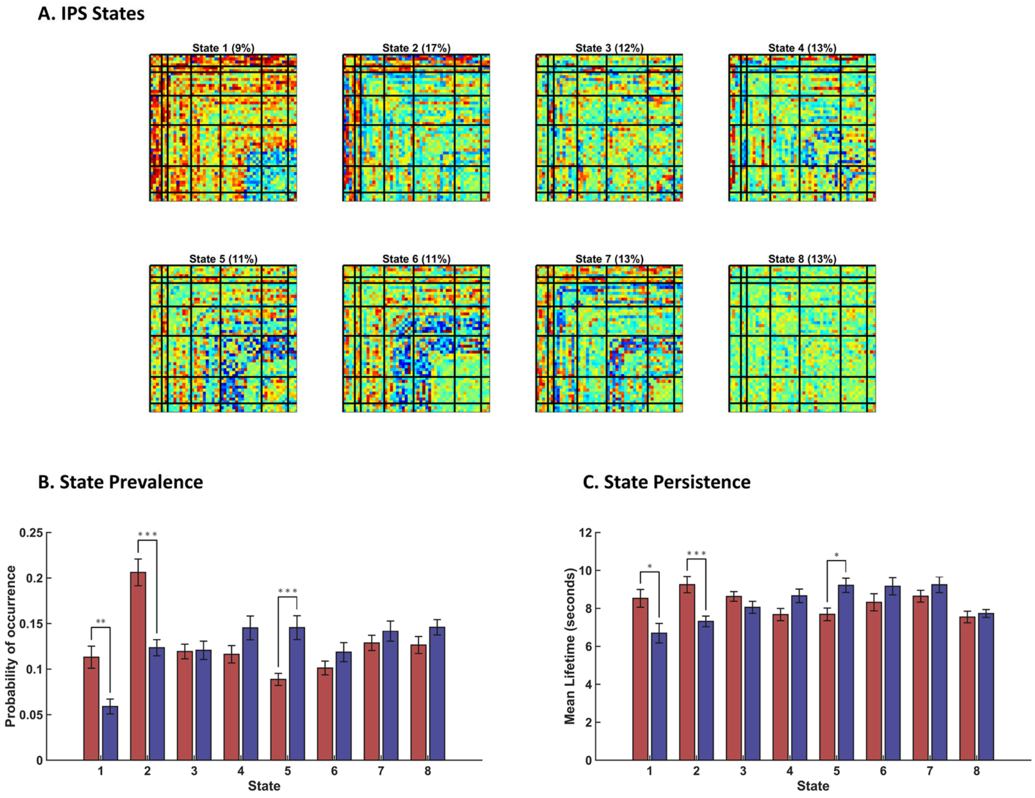

Figure S2: State characterization, for smoothing kernel with FWHM= 9 mm. (A) Instantaneous phase synchrony (IPS) states. The relative expression of each state (across time and subjects) is printed on top. (B) State prevalence. (C) State persistence. Error bars denote standard errors of mean. Asterisks show significant differences, after FDR correction (* stands for $p < 0.05$; ** stands for $p< 0.01$; *** stands for $p<0.001$).

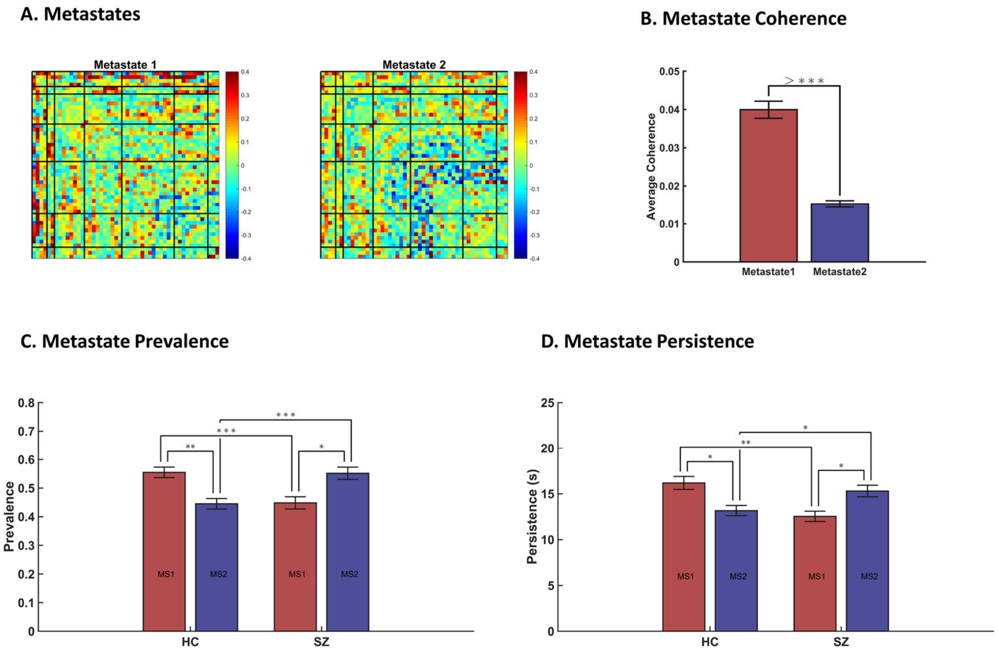

Figure S3: Metastate characterization, for smoothing kernel with FWHM= 9 mm. (A) Average metastate patterns. (B). Average phase coupling of metastates. (C) Prevalence of metastate 1 (MS1) and MS2. (D) Persistence of metastates. Differences were assessed using permutation-based t-tests (paired, when appropriate). Error bars denote standard errors of mean. Asterisks denote significant difference in mean, after FDR correction (* indicates $p < 0.05$; ** indicates $p< 0.01$; *** indicates $p<0.001$; >*** indicates $p<0.0001$).
